# Using host species traits to understand the consequences of resource provisioning for host–parasite interactions

**DOI:** 10.1111/1365-2656.12765

**Published:** 2017-11-13

**Authors:** Daniel J. Becker, Daniel G. Streicker, Sonia Altizer

**Affiliations:** ^1^ Odum School of Ecology University of Georgia Athens GA USA; ^2^ Center for the Ecology of Infectious Disease University of Georgia Athens GA USA; ^3^ Institute of Biodiversity, Animal Health and Comparative Medicine University of Glasgow Glasgow UK; ^4^ MRC‐University of Glasgow Centre for Virus Research Glasgow UK

**Keywords:** conservation, consumer–resource interactions, dietary breadth, home range, infectious disease, parasitism, phylogenetic meta‐analysis, supplemental feeding, urbanization

## Abstract

1. Supplemental food provided to wildlife by human activities can be more abundant and predictable than natural resources, and subsequent changes in wildlife ecology can have profound impacts on host–parasite interactions. Identifying traits of species associated with increases or decreases in infection outcomes with resource provisioning could improve assessments of wildlife most prone to disease risks in changing environments.
2. We conducted a phylogenetic meta‐analysis of 342 host–parasite interactions across 56 wildlife species and three broad taxonomic groups of parasites to identify host‐level traits that influence whether provisioning is associated with increases or decreases in infection.
3. We predicted dietary generalists that capitalize on novel food would show greater infection in provisioned habitats owing to population growth and food‐borne exposure to contaminants and parasite infectious stages. Similarly, species with fast life histories could experience stronger demographic and immunological benefits from provisioning that affect parasite transmission. We also predicted that wide‐ranging and migratory behaviours could increase infection risks with provisioning if concentrated and non‐seasonal foods promote dense aggregations that increase exposure to parasites.
4. We found that provisioning increased infection with bacteria, viruses, fungi and protozoa (i.e. microparasites) most for wide‐ranging, dietary generalist host species. Effect sizes for ectoparasites were also highest for host species with large home ranges but were instead lowest for dietary generalists. In contrast, the type of provisioning was a stronger correlate of infection outcomes for helminths than host species traits.
5. Our analysis highlights host traits related to movement and feeding behaviour as important determinants of whether species experience greater infection with supplemental feeding. These results could help prioritize monitoring wildlife with particular trait profiles in anthropogenic habitats to reduce infectious disease risks in provisioned populations.

## 1 INTRODUCTION

Resource availability affects population and community dynamics by affecting reproduction, dispersal and trophic interactions (Yang, Bastow, Spence, & Wright, 2008). These consumer–resource interactions are increasingly altered by human activities that subsidize wildlife. Food can be provided intentionally by bird feeders, tourism and conservation (Ewen, Walker, Canessa, & Groombridge, 2014; Robb, McDonald, Chamberlain, & Bearhop, 2008) and unintentionally by backyard gardens, agriculture and landfills (Gilbert et al., 2016; Streicker & Allgeier, 2016). Provisions can be more abundant and predictable than natural food and have persistent effects on animal demography, behaviour and physiology (Oro, Genovart, Tavecchia, Fowler, & Martínez‐Abraín, 2013).

Supplemental resources can also have profound impacts on host–parasite interactions (Murray, Becker, Hall, & Hernandez, 2016; Sorensen, van Beest, & Brook, 2013), in some cases increasing risks of cross‐species transmission (Becker, Streicker, & Altizer, 2015). For example, shifts in flying fox (*Pteropus* spp.) foraging behaviour towards mango farms were implicated in the spillover of Nipah virus into pigs and humans (Pulliam et al., 2012). However, provisioning can also reduce infection risk; for example, lace monitors (*Varanus varius*) feeding on urban garbage showed lower intensity of haemoparasites than naturally foraging hosts (Jessop, Smissen, Scheelings, & Dempster, 2012). These contrasting infection outcomes can partially be explained by the mechanisms by which supplemental feeding affects hosts (Becker & Hall, 2014, 2016). Resources can increase host fecundity, survival and aggregation, which can amplify parasite transmission (Cross, Edwards, Scurlock, Maichak, & Rogerson, 2007; Vicente, Höfle, Fernández‐De‐Mera, & Gortazar, 2007). However, greater resource access and better nutrition can also improve host immune defence by increasing pathogen clearance or resistance to infection, which can reduce transmission (Forbes et al., 2016; Wilcoxen et al., 2015). Understanding when these different mechanisms will dominate is thus important for predicting how provisioning will affect infection risks for wildlife, humans and domestic animals (Epstein et al., 2008; Lawson et al., 2012).

We here propose that host species traits may influence how provisioning affects infection (Table 1). Wildlife do not respond uniformly to increased food availability (Galbraith, Jones, Beggs, Parry, & Stanley, 2017; Marczak, Thompson, & Richardson, 2007). Many species that thrive in urban or agricultural habitats are food exploitation generalists (Kark, Iwaniuk, Schalimtzek, & Banker, 2007; Sih, Ferrari, & Harris, 2011), in that they consume a range of foods with varied nutritional values (Machovsky‐Capuska, Senior, Simpson, & Raubenheimer, 2016). Because generalists can exploit more novel foods, they often obtain greater population growth with provisioning (Bino et al., 2010; Prange, Gehrt, & Wiggers, 2003), which can increase transmission of density‐dependent parasites (McCallum, Barlow, & Hone, 2001). Generalists could also be exposed to more parasites in anthropogenic food (Brittingham, Temple, & Duncan, 1988; Sapolsky & Else, 1986) and be more susceptible to infections by foraging on poor‐quality or contaminated provisions (Birnie‐Gauvin, Peiman, Raubenheimer, & Cooke, 2017; Murray, Hill, Whyte, & Clair, 2016). However, generalist diets could also have reduced exposure to parasites with complex life cycles if omnivores shift their feeding towards anthropogenic food and away from intermediate hosts (Aponte et al., 2014; Hegglin, Bontadina, Contesse, Gloor, & Deplazes, 2007).

**Table 1:** Select host trait hypotheses for effects of resource provisioning on infection with microparasites, helminths and ectoparasites

Host trait	Effect on microparasites		Effect on helminths		Effect on ectoparasites	
Broad diet diversity	↑↑	Larger host densities increase contact, more exposure through food, malnutrition could increase host susceptibility	↓↓	Less exposure by switching to parasite‐free food, weaker effect of high host density	↑	Potential for higher density to increase transmission, weak effects on food exposure and susceptibility
Omnivory	↑↑	Larger host densities increase contact, more exposure through food, malnutrition could increase host susceptibility	↓↓	Less exposure by switching to parasite‐free food, weaker effect of high host density	↑	Potential for higher density to increase transmission, weak effects on food exposure and susceptibility
Fast pace of life	↑↓	Stronger fecundity response benefits host density, but improved adaptive immunity promotes recovery	↓	Weak effects of reproductive benefit, but enhanced adaptive immune defence	↑	Potential for higher density to increase transmission, but weak effects of stronger immunity
Large home range	↑↑	Contraction of home range promotes greater aggregation and contact rates	↑	Greater contact with infectious stages, but weak effect on complex life cycle parasites	↑	Dense aggregations promote close contact and free‐living exposure
Migratory	↑↑	Loss of migratory escape or culling, greater aggregation and contact rates	↑	Greater contact with infectious stages, but weak effect on complex life cycle parasites	↑	Loss of migratory escape or culling, greater aggregation and contact rates

Wildlife species that exploit anthropogenic resources or that receive supplemental food from conservation programmes also vary in their life‐history traits (McKinney, 2006; Møller, 2009). Species that invest more in reproduction (i.e. *r*‐selected) could show stronger demographic responses to provisioning. For example, multi‐brood birds advance their egg‐laying date more so than single‐brood birds in response to supplemental feeding (Dhondt, 2010; Svensson, 1995); this could potentially increase population sizes and the prevalence of parasites transmitted by close contact. Differential immune investment between fast‐ and slow‐lived species could also influence infection outcomes (Lee, 2006). Animals with fast life histories tend to allocate less energy towards parasite resistance and adaptive immunity, because the developmental costs of these defences exceed their fitness benefits for short‐lived species (Cronin, Welsh, Dekkers, Abercrombie, & Mitchell, 2010; Previtali et al., 2012). As reduced starvation enhances these defences (Martin, Navara, Weil, & Nelson, 2007), provisioning could reduce physiological trade‐offs (Brzęk & Konarzewski, 2007) and enhance adaptive immunity for *r*‐selected species. Although increased resistance and population size in fast‐lived species could have weak effects on parasites spread by close contact, improved resistance could have stronger effects on parasites for which transmission is divorced from host density.

Movement and ranging behaviour might also moderate the relationship between provisioning and infection, as supplemental resources can promote dense host aggregations and encourage sedentary behaviour (Corcoran et al., 2013; Gilbert et al., 2016). Species that naturally forage over large areas could experience increased infection if provisioning contracts host home ranges and thus promotes contact with infected conspecifics or build‐up of parasite infectious stages in the environment (Hines, Ezenwa, Cross, & Rogerson, 2007; Wright & Gompper, 2005). Shifts from migratory to sedentary behaviour driven by supplemental food that is available year‐round could also increase infection by eliminating ecological mechanisms such as migratory escape and migratory culling that reduce infection risk (Altizer, Bartel, & Han, 2011; Hall, Altizer, & Bartel, 2014).

In this study, we performed a phylogenetic meta‐analysis to test effects of host dietary breadth, trophic level, pace of life, home range and migratory status on infection outcomes of resource provisioning. We collated 342 published relationships between supplemental feeding and parasitism across 61 studies and paired standardized effect sizes with trait data for 56 host species spanning mammals, amphibians, fish, reptiles and birds. We assessed phylogenetic dependence in effect sizes (i.e. the possibility that infection risks of closely related host species respond similarly to provisioning) and used phylogenetic metaregression to identify ecological correlates of effect sizes while controlling for host phylogeny, clustering of effect sizes within studies and sampling variance. We stratified data by infection with microparasites (i.e. bacteria, viruses, protozoa, fungi), helminths and ectoparasites to account for previously observed differences in effect sizes based on parasite biology (Becker et al., 2015) and to test hypothesized interactions with host traits (Table 1). By accounting for effects of host and parasite biology, we therefore provide important steps to establish a framework for predicting which wildlife species experience greater infection risks with resource provisioning and supplemental feeding.

## 2 MATERIALS AND METHODS

### 2.1 Effect size data

We collected data on the relationships between provisioning and infection (by any class of pathogen or parasite, including viruses, bacteria, protozoa, fungi, helminths and ectoparasites) in wild vertebrates from studies identified through a systematic search of Web of Science, Google Scholar, PubMed and CAB Abstracts in 2014 (Becker et al., 2015). We expanded this previous dataset, which contained 132 records spanning 23 studies and 16 host species, by performing the same search from 2014 through 2016, adding an additional 132 host–parasite interactions from 20 studies and 30 host species. We also extracted data from 19 references cited within these publications, providing an additional 78 records from 19 species and resulting in a total dataset of 342 records from 56 hosts and 61 studies (Appendix S1, Table S1, Figure S1); a list of all included studies is provided in the “Data Sources” section. We included captive studies that used mesocosms or where hosts were first caught in the wild (e.g. Knutie, Wilkinson, Wu, Ortega, & Rohr, 2017). Infection outcomes included binary infection status, prevalence, intensity and seroprevalence as functions of feeding treatment or gradients of anthropogenic food. For each host–parasite interaction, we recorded the host species, parasite species and type (microparasite, helminth, ectoparasite), provisioning type (intentional or accidental), sample size, test statistics and directional effect of provisioning on infection (i.e. increased or decreased). Our dataset had 145 records for microparasites, 135 records for helminths and 62 records for ectoparasites. Infection was mostly measured as binary infection status or prevalence across our dataset (74%) and within each parasite group (microparasites: 76%, helminths: 73%, ectoparasites: 73%). We assessed the main transmission routes of each parasite with the Global Mammal Parasite Database (Stephens et al., 2017). In the 259 records for which these data were available, most microparasites were transmitted by close contact and non‐close contact (79%) while most helminths were transmitted by non‐close contact and intermediate hosts (94%); in contrast, all ectoparasites were transmitted by close contact or non‐close contact.

We converted test statistics into correlation coefficients (Rosenthal & DiMatteo, 2001). We followed Wolf (1986) and Borenstein, Hedges, Higgins, and Rothstein (2009) for converting *X* ^2^ and *F* statistics into *r*. When effects were presented as odds ratios, we applied Digby's approximation (Bonett, 2007). When test statistics were not reported, we either contacted authors, simplified prevalence and seroprevalence to a contingency table and calculated *X* ^2^, or calculated the standardized mean difference (Cohen's *d*) for intensity outcomes. We assigned negative values to correlations where infection outcomes were lower in provisioned populations and converted directional *r* into Fisher's *Z* (*Zr*) as a stabilizing transformation (Borenstein et al., 2009). Positive values indicate increased infection in provisioned wildlife, while negative scores indicate decreased infection. We used the r package *metafor* to convert effect sizes (R Core Team, 2013; Viechtbauer, 2010).

### 2.2 Host species trait data

**Figure 1:**
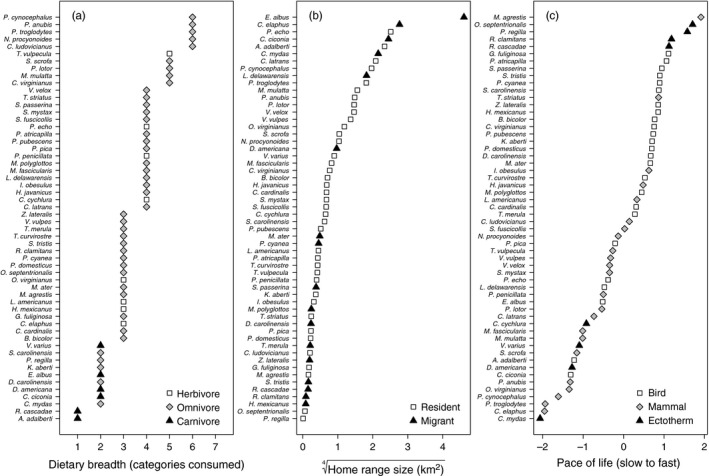
Distribution of trait covariates based on species feeding behaviour (a), movement ecology (b) and the first phylogenetic PC for pace of life covariates (c), representing an axis of slow to fast life histories. Galapagos finches were standardized as *Geospiza fuliginosa*

To test how species‐specific traits influence effects of provisioning on parasites (Table 1), we collected data on host dietary breadth, trophic level, pace of life, home range and migratory status (Figure 1). Dietary breadth was defined as the number of food categories consumed per species and was standardized by the PanTHERIA database of mammal traits: vertebrate, invertebrate, fruit, flowers/nectar/pollen, leaves/branches/bark, seeds, grass and roots/tubers (Jones et al., 2009). For trophic level, we defined hosts as herbivores, omnivores or carnivores. Pace of life covariates included adult body mass, adult body size, offspring per year, maximum life span and age at sexual maturity. Because of high correlation in these traits (Appendix S2, Table S1, Figure S2), we performed a phylogenetic principal component (PC) analysis, which accounts for closely related species sharing similar trait values using the phytools package (Revell, 2009, 2012). The first two phylogenetic PCs accounted for 79% of the variation in life history, with PC1 (58% of variation) loaded negatively by log mass (−0.87), square‐root body size (−0.85), log age of sexual maturity (−0.84) and log life span (−0.78) and positively by log offspring per year (0.33). Large, positive PC1 values thus indicate hosts categorized along the fast pace of life continuum (i.e. *r‐*selected), investing more in early reproduction at the expense of body size and life span (Appendix S2, Figure S3). Home range size was defined as the area (km^2^) in which daily activities are restricted (Jones et al., 2009) and was normalized with a quarter‐root transformation. Lastly, we defined a species as migratory if its movement behaviour tracks seasonal changes in resources, mates or habitat (Dingle, 2014).

For mammals (*n* = 23), trait data were derived from PanTHERIA. For the 33 non‐mammals and for mammals where trait values were missing, data were derived from AnAge (http://genomics.senescence.info/species/), Animal Diversity Web (ADW) at the University of Michigan Museum of Zoology (http://animaldiversity.org/), ARKive (http://www.arkive.org/) or the primary literature. As we observed discrepancies between PanTHERIA dietary data and known ecology (e.g. omnivorous *Papio cynocephalus* was defined as herbivorous), we systematically revised dietary breadth and trophic level for all mammals. Additional details are provided in the Supporting Information (Appendix S3, Table S2). As dietary breadth is a discrete trait, we categorized species as having low (1–2), medium (3–4) or high (5–6) dietary breadth.

### 2.3 Assessing phylogenetic signal

**Figure 2:**
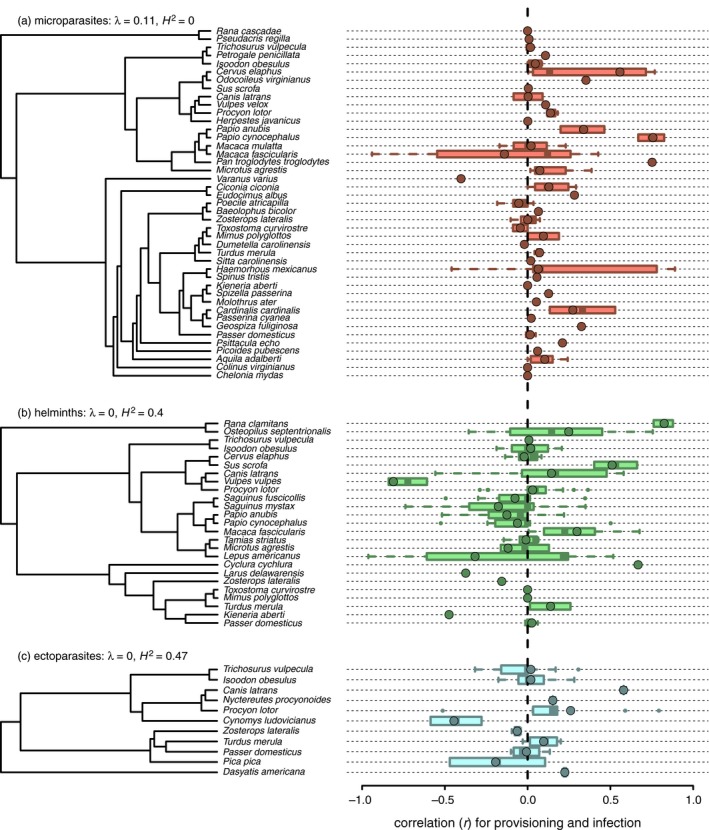
Phylogenetic visualization of infection outcomes of resource provisioning for microparasites (a), helminths (b) and ectoparasites (c). Boxplots show the median and first and third quartile of effect sizes (back‐transformed *r*), whiskers show the range of non‐outliers and open circles show potential outliers. Filled circles display the weighted mean effect sizes per host species. Legends display estimates of Pagel's λ and phylogenetic heritability (*H* ^2^) in effect sizes

We used two methods to assess whether closely related host species had more similar effect sizes. For both methods, we obtained a phylogeny from the Open Tree of Life using the rotl package (Hinchliff et al., 2015; Michonneau, Brown, & Winter, 2016); the ape package was used to prune the tree to our host species, to resolve multichotomies, and to provide branch lengths by converting the tree to ultrametric format using Grafen's method (Grafen, 1989; Paradis, Claude, & Strimmer, 2004). First, we calculated phylogenetic signal in mean effect sizes per host species per parasite group. Within the datasets for microparasites (*n* = 43 hosts), helminths (*n* = 25 hosts) and ectoparasites (*n* = 11 hosts), 52%, 80% and 82% of hosts had more than one effect size, and the number of records per host ranged from 1 to 22 for microparasites (μ = 3.3), 1 to 28 for helminths (μ = 5.4), and 1 to 20 for ectoparasites (μ = 5.6). For species with multiple effect sizes, we derived the average *Zr* effect sizes by weighting each observation by its sample size. We then used maximum likelihood (ML) and the caper package to estimate Pagel's λ in mean effect sizes for each parasite group (Orme, 2013; Pagel, 1999). We compared our ML estimates of λ against models of no phylogenetic dependence (λ = 0) and a Brownian motion model of evolution (λ = 1) using likelihood ratio tests. As calculating weighted species averages risks losing information on the within‐study and within‐species variance in effect sizes across parasites (Nakagawa & Santos, 2012), we also assessed phylogenetic signal in effect sizes by fitting hierarchical random‐effects meta‐analysis models (REMs) with observation, study, and species set as random effects (Konstantopoulos, 2011). Study was included as a random effect given that most studies (36/61) contained more than one effect size. For each REM, the covariance structure of the species random effect was specified by the correlation matrix of our host phylogeny (Bentz, Becker, & Navara, 2016). We fit REMs using restricted ML (REML) to obtain unbiased estimates of variance components, from which we calculated phylogenetic heritability (*H* ^2^; Housworth, Martins, & Lynch, 2004; Nakagawa & Santos, 2012). *H* ^2^ is analogous to Pagel's λ in that *H* ^2^ = 1 corresponds to strong phylogenetic dependence in effect sizes. REMs were fit with the rma.mv function in the metafor package (Viechtbauer, 2010) and used Broyden–Fletcher–Goldfarb–Shanno (BFGS) optimization to improve convergence. The distribution of effect sizes per species and across parasite groupings is displayed in Figure 2 with estimates of Pagel's λ and *H* ^2^.

### 2.4 Testing host traits correlates on provisioning effect sizes

We next used phylogenetic metaregression to identify host trait correlates of effect sizes while accounting for unit‐level heterogeneity, study pseudoreplication and phylogeny. We included observation, study and species as random effects in mixed‐effects models (MEMs) fit separately to each parasite group dataset. We considered univariate MEMs of each trait, biologically meaningful interactions between traits where collinearity was weak and that were supported by the sample size, and interactions between traits and supplemental feeding type (Appendix S4, Tables S3 and S4). We ignored the interaction between home range size and pace of life as home range scales with body size (McNab, 1963). A candidate set of all possible additive MEMs given these restrictions and excluding collinear traits was generated for each parasite dataset with the mumin package (Bartoń, 2013). All sets included an intercept‐only model and were limited to at most three covariates per model to keep the number of MEMs low relative to the sample size.

We first fit MEMs with ML to allow model comparison using Akaike information criterion corrected for small sample size (AIC_c_; Burnham & Anderson, 2002). We next calculated variable importance as the summed Akaike weights (*w*_*i*_) for all MEMs in which a given predictor occurred. We refit MEMs with REML to obtain unbiased estimates of the random effects and calculate three pseudo‐*R* ^2^: the proportional reduction in the summed variance components of each MEM compared with the summed variance components of a model without predictors (i.e. the REM) (Brace et al., 2017; $R_{v}^{2}$), the proportional reduction in residual variance of each MEM compared with the residual variance of a model without predictors (i.e. the REM) (Xu, 2003; $R_{r}^{2}$), and the adjusted *R* ^2^ from a weighted linear regression of observed vs. predicted effect sizes ($R_{p}^{2}$). All models were again fit with the rma.mv function in the metafor package (Viechtbauer, 2010) and used BFGS optimization. We considered models within two ΔAIC_c_ to be competitive and visualized top MEMs by back‐transforming *Zr* into correlation coefficients (*r*) for interpretability and classified results into trivial (<.1), small–medium (.1–.3), medium–large (.3–.5) and large–very large (>.5) effects (Cohen, 1992).

## 3 RESULTS

### 3.1 Effect size range and phylogenetic signal

The effect of provisioning on infection varied widely among hosts (Figure 2). Species that displayed the greatest mean increase in infection with provisioning included *Papio cynocephalus*,* Pan troglodytes* and *Cervus elaphus* for microparasites (*r* = .76, .75, .56); *Rana clamitans*,* Cyclura cychlura* and *Sus scrofa* for helminths (*r* = .82, .67, .51); and *Canis latrans* for ectoparasites (*r* = .58). Species that displayed the greatest mean decrease in infection with provisioning included *Varanus varius* for microparasites (*r* = −.49); *Vulpes vulpes*,* Kieneria aberti* and *Larus delawarensis* for helminths (*r* = −.81, −.47, −.37); and *Cynomys ludovicianus* for ectoparasites (*r* = −.45). Average effect sizes were weakly related to host phylogeny and depended on parasite group (Figure 2). Likelihood ratio tests suggested all estimates of Pagel's λ differed from Brownian motion models of evolution (*p* ≤ .01) and did not differ from models of phylogenetic independence (*p* ≥ .33). However, estimates of phylogenetic signal differed when accounting for within‐study and within‐species variance in effect sizes. While *H* ^2^ for microparasites was 0, helminths and ectoparasites showed moderate phylogenetic dependence in effect sizes (*H* ^2^ = 0.40 and 0.46 respectively).

### 3.2 Trait correlates of effect sizes for microparasites

Species traits were generally more important predictors of the relationship between provisioning and infection with microparasites than the provisioning type (Table 2). The top trait predictors of effect sizes with microparasites were home range (importance = 0.70), dietary breadth (0.47), pace of life (0.39) and migratory status (0.27); provisioning type had relative importance of 0.19.

**Table 2:** Relative variable importance (%) for all predictors in the phylogenetic metaregression models (MEMs) for effect sizes with microparasites, helminths and ectoparasites. MEM, mixed‐effects model

Predictors	Microparasite MEMs	Helminth MEMs	Ectoparasite MEMs
Dietary breadth	0.47	0.00	1.00
Dietary breadth × pace of life	0.04	0.00	NA
Dietary breadth × home range size	0.11	0.00	0.00
Migratory status	0.27	0.00	0.00
Pace of life	0.39	0.60	0.00
Pace of life × migratory status	0.00	0.00	NA
Provisioning type	0.19	0.99	0.32
Provisioning type × dietary breadth	0.00	0.00	0.00
Provisioning type × migratory status	0.00	NA	NA
Provisioning type × pace of life	0.00	0.00	0.00
Home range size × provisioning type	0.00	0.00	NA
Provisioning type × trophic level	0.00	NA	NA
Home range size	0.70	0.18	1.00
Home range size × migratory status	0.00	0.00	NA
Trophic level	0.05	0.48	0.00
Trophic level × migratory status	0.00	NA	NA
Trophic level × pace of life	0.00	0.00	NA

**Figure 3:**
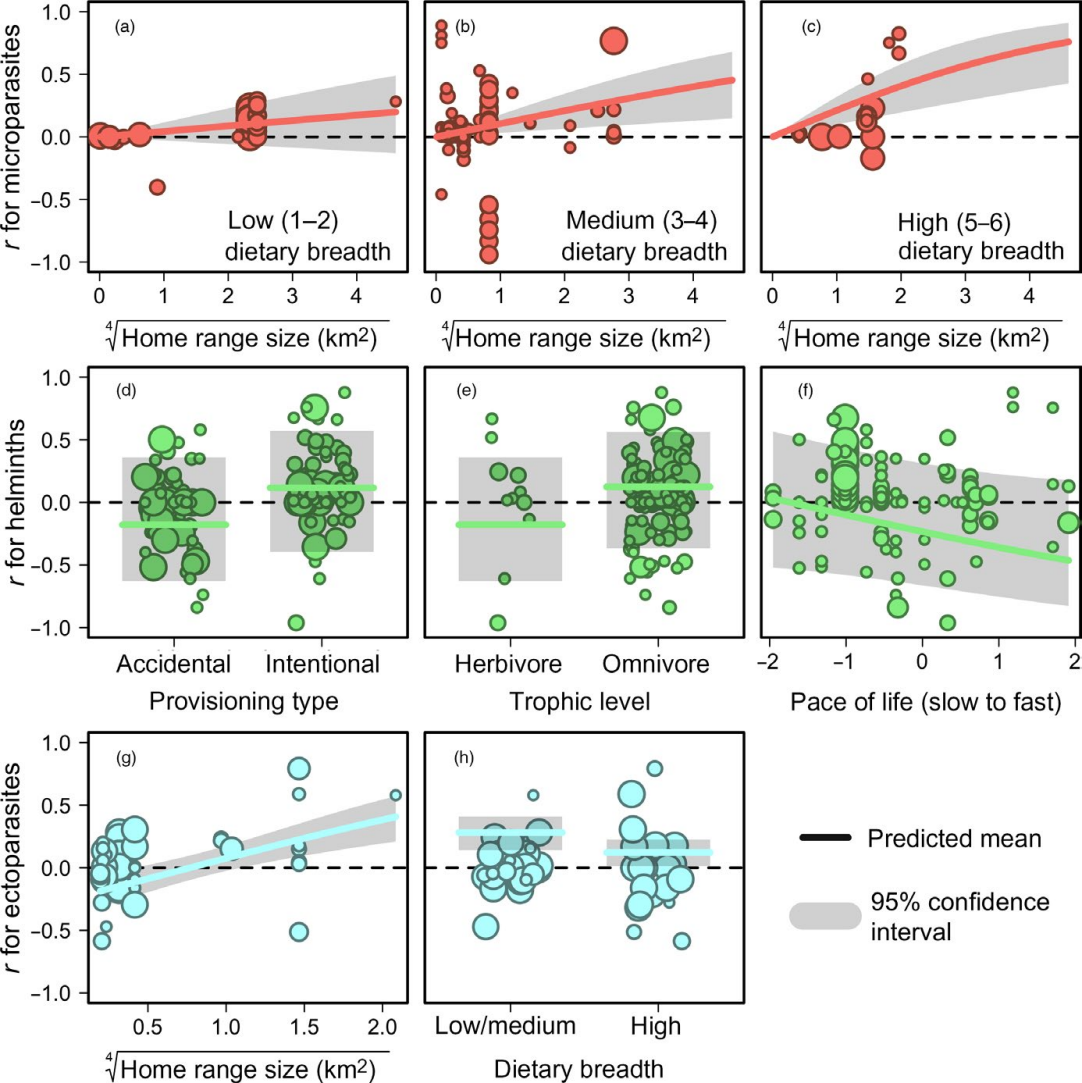
Competitive mixed‐effects models (MEMs) correlating trait and supplemental feeding predictors to effect sizes (back‐transformed *r*) for microparasites, helminths and ectoparasites, with points scaled by the inverse sampling variance. Predicted means and 95% confidence intervals are shown with solid lines and bands. The dashed line shows where *r* = 0 (i.e. provisioning has no effect on infection)

Comparison of 43 candidate models for explaining microparasite effect sizes found seven MEMs to be within two ΔAIC_c_ (Table 3 and Table S5). A univariate MEM with home range size was the most supported (ΔAIC_c_ = 0.00, *w*_*i*_ = 0.12); this covariate was present in most of the top MEMs (6 of 7) and explained up to 11% of the variation in effect sizes, with wide‐ranging host species showing the largest positive effect sizes (*Q*_*M*_ = 4.62, *df* = 1, *p* = .03). Models with interactive and additive relationships between home range size and dietary breadth were also competitive (ΔAIC_c_ = 0.26–0.42, *w*_*i*_ = 0.11–0.10). The interactive MEM explained up to 14% of the variation in effect sizes (*Q*_*M*_ = 13.23, *df* = 5, *p* = .02) and showed that hosts with low dietary breadth had no relationship between home range and effect sizes (β = 0.04, *z* = 1.19, *p* = .23; Figure 3a) while hosts with medium and high dietary breadth showed a strong positive relationship (all β ≥ 0.11, *z* ≥ 2.82, *p* < .01; Figure 3b,c), suggesting that dietary generalists that also have large home ranges may be most prone to greater microparasite risks with provisioning. The remaining competitive models also contained pace of life and migratory status (ΔAIC_c_ = 1.22–1.90), but had lower support (*w*_*i*_ = 0.07–0.05); these MEMs suggested trends for microparasites to increase with provisioning in fast‐lived (β = 0.03, *z* = 0.47, *p* = .64) and migratory hosts (β = 0.07, *z* = 0.96, *p* = .34), but coefficients did not differ from zero.

**Table 3:** Ranking of mixed‐effects models (MEMs) predicting infection outcomes of provisioning for microparasites, helminths and ectoparasites. Models are ranked by ΔAIC_c_ alongside the Akaike weights (*w*_*i*_), residual phylogenetic signal (*H* ^2^), number of parameters (*k*) and pseudo‐*R* ^2^ statistics ($R_{v-p}^{2}$). Only MEMs within two ΔAIC_c_ are shown (see Appendix S5 for the ranking of all MEMs)

	ΔAIC_c_	*w*_*i*_	*H* ^2^	*k*	$R_{v}^{2}$	$R_{r}^{2}$	$R_{p}^{2}$
*Microparasite outcomes*							
*Zr* ~ home range	0.00	0.12	0.00	2	0.08	0.02	0.11
*Zr* ~ dietary breadth × home range	0.26	0.11	0.00	6	0.14	0.08	0.09
*Zr* ~ dietary breadth + home range	0.42	0.10	0.00	4	0.11	0.05	0.11
*Zr* ~ dietary breadth + home range + pace of life	1.22	0.07	0.00	5	0.12	0.06	0.13
*Zr* ~ dietary breadth + home range + migratory status	1.30	0.06	0.00	5	0.13	0.08	0.13
*Zr* ~ pace of life	1.45	0.06	0.00	2	0.03	0.00	0.01
*Zr* ~ home range + migratory status	1.90	0.05	0.00	3	0.08	0.03	0.13
*Helminth outcomes*							
*Zr* ~ pace of life + provisioning type + trophic level	0.00	0.16	0.66	4	0.00	0.04	0.23
*Zr* ~ provisioning type	0.74	0.11	0.51	2	0.00	0.06	0.10
*Zr* ~ provisioning type + trophic level	0.83	0.11	0.66	3	0.00	0.08	0.16
*Zr* ~ pace of life + provisioning type + home range	0.96	0.10	0.42	4	0.08	0.09	0.25
*Zr* ~ pace of life + provisioning type	1.63	0.07	0.48	3	0.00	0.06	0.15
*Ectoparasite outcomes*							
*Zr* ~ dietary breadth + home range	0.00	0.29	0.00	3	0.52	0.28	0.14
*Zr* ~ dietary breadth + home range + provisioning type	1.49	0.14	0.00	4	0.50	0.29	0.14

### 3.3 Trait correlates of effect sizes for helminths

In contrast to effect sizes for microparasites, provisioning type was the strongest predictor of effect sizes for helminths with relative importance of 0.99 (Table 2). Top host trait predictors included pace of life (0.60), trophic level (0.48) and home range size (0.18); in contrast, both migratory status and dietary breadth were not important predictors (relative importance = 0).

Comparison of 43 candidate models for explaining helminth effect sizes found five MEMs to be within two ΔAIC_c_ (Table 3 and Table S6). An additive MEM of provisioning type, pace of life, and trophic level was the most competitive (ΔAIC_c_ = 0.00, *w*_*i*_ = 0.16) and explained up to 23% of the variation in effect sizes (*Q*_*M*_ = 12.32, *df* = 3, *p* < .01). This MEM showed effect sizes to be larger and more positive for intentional compared to unintentional provisioning (β = 0.30, *z* = 3.18, *p* = .001; Figure 3d) and for omnivores than herbivores (β = 0.31, *z* = 1.93, *p* = .05; Figure 3e); pace of life was also marginally negatively related to effect sizes (β = −0.14, *z* = 1.62, *p* = .11), suggesting fast‐lived species to have lower helminth infection (Figure 3f). Yet this model received close support to a univariate MEM of provisioning type (ΔAIC_c_ = 0.74, *w*_*i*_ = 0.11), which also appeared in all competitive MEMs, suggesting results were driven mostly by this predictor. An additive model with home range received equivalent support (ΔAIC_c_ = 0.96, *w*_*i*_ = 0.10) and showed a marginal negative relationship with effect sizes (β = −0.23, *z* = 1.57, *p* = .12).

### 3.4 Trait correlates of effect sizes for ectoparasites

For effect sizes with ectoparasites, dietary breadth and home range size had 100% relative importance (Table 2), while that of only provisioning type differed from zero (0.32). Comparison of 39 candidate MEMs for ectoparasite effect size identified only two models within two ΔAIC_c_ (Table 3 and Table S7). An additive MEM of home range size and dietary breadth was the most supported (ΔAIC_c_ = 0.00, *w*_*i*_ = 0.29) and explained up to 52% of the variation in effect sizes (*Q*_*M*_ = 16.99, *df* = 2, *p* < .001). This model showed that effect sizes increased with species home range size (β = 0.33, *z* = 4.12, *p* < .001; Figure 3g) and were larger for species with low and medium dietary diversity compared to species with high dietary diversity (β = 0.17, *z* = 2.28, *p* = .02; Figure 3h). The other MEM contained both traits and provisioning type (ΔAIC_c_ = 1.49, *w*_*i*_ = 0.14), although effect sizes did not differ between feeding contexts (β = −0.10, *z* = 1.16, *p* = .25).

## 4 DISCUSSION

Our analysis shows that host species traits related to movement ecology and feeding behaviour are important predictors of how resource provisioning correlates with parasitism in wildlife. Dietary generalists with large home ranges demonstrated the strongest increases in infection with microparasites under provisioning. Similarly, wide‐ranging species had higher prevalence and intensity of ectoparasites with supplemental feeding, although effect sizes were also high for dietary specialists. For infection with helminths, the type of provisioning was the most important determinant of effect sizes; however, we also found weaker effects of host trophic level on infection outcomes. These results more broadly support the hypothesis that host traits can mediate how supplemental resources affects parasite transmission and provide a framework for predicting which wildlife species experience greater infection risks with resource provisioning.

### 4.1 Predictors of microparasite infection

Host species with intrinsically large home ranges had the greatest increase in infection with microparasites in provisioned habitat. Species with large home ranges tend to have small population densities and therefore could naturally experience low exposure to parasites spread by close and non‐close contact (Bordes, Morand, Kelt, & Van Vuren, 2009; Han, Park, Jolles, & Altizer, 2015). As supplemental feeding can restrict home range size and promote sedentary behaviour, artificially increased aggregation for naturally wide‐ranging, low‐density species could have stronger effects on increasing exposure to directly and environmentally transmitted microparasites than for species that naturally live at high densities and inhabit small geographic areas. For example, winter feeding of elk (*Cervus elaphus*), which have large home ranges, promotes dense host aggregations that elevate contact rates and brucellosis transmission (Cross et al., 2007). Our analyses thus more generally support the hypothesis that naturally wide‐ranging species experience high infection with microparasites with provisioning owing to contraction of home ranges and increased contact rates.

Model comparison also illustrated that the association between home range size and microparasite risk was most pronounced for dietary generalists. Species with larger home ranges and broad dietary breadth, such as coyotes (*Canis latrans*), baboons (*Papio anubis* and *cynocephalus*) and raccoons (*Procyon lotor*) showed the greatest increase in infection in provisioned habitat. However, microparasite infection in species with wider home ranges but narrow dietary breadth, such as green sea turtles (*Chelonia mydas*), white storks (*Ciconia ciconia*), and Spanish imperial eagles (*Aquila adalberti*), showed weaker responses to provisioning. Greater dietary breadth could enhance microparasite transmission in provisioned habitat if it facilitates exposure to bacteria and viruses within anthropogenic food (e.g. Sapolsky & Else, 1986), reliance on poor‐quality or contaminated foods that promote malnutrition and weaker immune defence (e.g. Becker et al., 2017), or increases in population size and thus density‐dependent transmission (e.g. Prange et al., 2003). Such mechanisms could have strong effects on these wide‐ranging species given that theory predicts elevated contact rates (e.g. from contracted home range), population density (e.g. from capitalizing on novel foods) and susceptibility to infection (e.g. from switching to poor‐quality food) from provisioning should produce net increases in microparasite prevalence (Becker & Hall, 2014; Becker et al., 2015).

Host migration status and trophic level did not appear to influence infection responses to provisioning. This could reflect a dominance of resident (29/43) and omnivorous (32/43) species in this dataset, limiting power to detect effects for herbivores and carnivores that practice migration or nomadic behaviour and for which provisioning produces year‐round food resources. Yet many herbivores such as elk (*Cervus elaphus*) and mule deer (*Odocoileus hemionus*) have delayed spring migration or migrate shorter distances in response to supplemental feeding (Jones et al., 2014; Peterson & Messmer, 2007). Similarly, naturally nomadic Australian white ibis (*Threskiornis molucca*) have become more sedentary owing to food provided by landfills (Coogan et al., 2017; Martin, French, & Major, 2010), which could explain positive associations between provisions from urban parks in Florida and the prevalence of *Salmonella* in white ibis (*Eudocimus albus*) (Hernandez et al., 2016). To better address if provisioning‐mediated loss of migration elevates infection risk, future work could expand the taxonomic breadth of this test by examining infection between provisioned and unprovisioned hosts in other migratory or nomadic species that capitalize on urban food sources, such as *Pteropus* fruit bats (Plowright et al., 2015).

### 4.2 Predictors of ectoparasite infection

As with our findings for microparasites, species with large home ranges also had the greatest increase in prevalence or intensity with ectoparasites in provisioned habitat. For example, wide‐ranging hosts such as raccoons (*Procyon lotor*) and southern stingrays (*Dasyatis americana*) showed larger, positive effect sizes, and the increased aggregations of such species have been associated with greater contact rates and transmission of multiple ectoparasites (Monello & Gompper, 2010; Semeniuk & Rothley, 2008). As all ectoparasites in our study are transmitted by close or non‐close contact, this finding again supports the idea that contraction of home range for wide‐ranging species under supplemental feeding can artificially inflate host aggregations and opportunities for ectoparasite transmission. Because this trait received strong support on its own (i.e. not in an interaction), this finding suggests home range size may be a more general predictor of infection outcomes for ectoparasites than for microparasites. This could arise from ectoparasites focusing questing behaviour towards areas with dense host populations (Burg, 2001).

In contrast to our results for microparasites and to our original predictions for this parasite group, provisioning increased ectoparasite outcomes slightly more for dietary specialists than for dietary generalists. Biological explanations for this result remain unclear, given that species with specialized diets would be expected to experience less dramatic population growth with provisioning compared to generalists (Prange et al., 2003). Furthermore, these results are difficult to properly interpret given that our ectoparasite data were dominated by omnivorous hosts (9 of 11), with four species showing true dietary generalism (five to six food items) and remaining species showing mostly moderate dietary breadth (three to four food items). Only southern stingrays (*Dasyatis americana*) were classified as having low dietary breadth (≤2 food items). Future studies examining patterns of ectoparasitism in commonly provisioned dietary specialists such as vultures (Cortés‐Avizanda et al., 2016) would help assess the generality of this finding.

### 4.3 Predictors of helminth infection

While we identified home range size and dietary breadth as key correlates for how microparasite and ectoparasite outcomes respond to provisioning, neither trait explained variation in helminth outcomes. This was unexpected, as the same mechanisms of contracted home ranges and sedentary behaviour could also increase exposure to helminths transmitted by direct contact or environmental infectious stages (Hines et al., 2007). One explanation could be that helminths with complex life cycles, common in our dataset, might not respond to increases in host aggregation. A subsequent prediction could be that hosts with broad dietary breadth have lower exposure to helminths under provisioning, as this trait could facilitate switching diets away from natural intermediate hosts and towards anthropogenic resources free of infection (Hegglin et al., 2007). Yet, we found evidence that omnivores show greater helminth infection with provisioning than herbivores. While this could imply support for the dietary mechanisms observed for microparasites (e.g. omnivores obtaining higher densities or being more prone to malnutrition), our dataset was again dominated by omnivores (21 of 25), suggesting studies of herbivores and especially carnivores are needed to understand consequences of provisioning for helminths.

In contrast to the results for microparasites and ectoparasites, the context of supplemental feeding was a more important predictor of effect sizes for helminths than host traits. This corroborates findings from an earlier meta‐analysis (Becker et al., 2015), demonstrating that the prevalence and intensity of helminths are greater when wildlife are intentionally provisioned. Our result across a larger sample of hosts and parasites better supports the idea that supplemental feeding based on wildlife management, recreational feeding and tourism can promote build‐up of environmental infectious stages and enhance susceptibility from poor‐quality foods that do not match natural diets (Murray, Becker, et al., 2016). Furthermore, this finding suggests that such risks for helminth infection may be more general and thus applicable across host taxa.

### 4.4 Applications to human health and wildlife conservation

Host traits collectively explained between 14% and 52% of the variation in how provisioning affects infection with microparasites and ectoparasites, while supplemental feeding context remained a stronger predictor of helminth outcomes. While this finding highlights further work is necessary to understand divergent infection outcomes from provisioning, particularly with helminths and to a certain extent microparasites, our analysis also provides an important step for predicting which species are prone to greater infection with microparasites and ectoparasites with anthropogenic resource shifts. More generally, comparative analyses have identified groups and traits of wildlife that host an unusually high number of zoonotic pathogens (Han, Kramer, & Drake, 2016). Here, our models suggest dietary generalists with large home ranges are prone to greater infection by microparasites with provisioning, whereas wide‐ranging dietary specialists are more likely have greater prevalence and intensity of ectoparasites. As an application of these conclusions, Jamaican fruit‐eating bats (*Artibeus jamaicensis*) consume a range of fruit, nectar, pollen, flowers and even insects (Heithaus, Fleming, & Opler, 1975), which may explain their ability to capitalize on food provided by agricultural crops and backyard trees (Bolívar‐Cimé, Laborde, MacSwiney, & Sosa, 2015). As this species is also thought to have a moderately large home range (Fleming, 1992), this combination of traits would predict bats foraging in provisioned habitats to have higher odds of microparasite infection than those in unprovisioned habitats. As this species can be infected with zoonoses such as rabies virus (Reid & Jackson, 2001), higher infection prevalence in urban‐ and agriculture‐foraging populations could increase the likelihood of pathogen spillover (Plowright et al., 2017). Targeted surveillance of species with similar trait profiles and in close contact with humans could help predict and manage these infectious disease risks.

From another perspective, supplemental feeding is often proposed as a conservation tool for threatened populations (Ewen et al., 2014) or to limit human–wildlife conflict (Kubasiewicz, Bunnefeld, Tulloch, Quine, & Park, 2015). Our findings suggest these practices could most benefit dietary specialists with small home ranges when considering risks of microparasite infection. For example, supplemental feeding has been used to reverse declines of endangered Iberian lynx (*Lynx pardinus*) in Spain (López‐Bao, Palomares, Rodríguez, & Delibes, 2010). Owing to the narrow dietary breadth of lynx (i.e. obligate carnivores) and their smaller home range size (Jones et al., 2009), our models predict this practice may not increase infections that are considered threats to population viability, such as feline leukaemia virus (Meli et al., 2009); however, trade‐offs could exist with promoting higher ectoparasite burdens. Supplemental feeding is also practiced to limit human–brown bear (*Ursus arctos*) conflicts in urban habitats (Huber, Kusak, Majić‐Skrbinšek, Majnarić, & Sindičić, 2008). Yet, in contrast to the lynx example, our models predict that provisioning of this wide‐ranging, generalist species (Jones et al., 2009) could instead amplify the transmission of microparasites such as West Nile virus and canine parvovirus (Madić, Huber, & Lugović, 1993); subsidized bears could both experience poorer health and serve as reservoir hosts. Such predictions could motivate managers to limit supplemental feeding or enhance existing practices for such species with “risky” trait profiles by providing nutritionally complete diets and spatially dispersed feeding stations to reduce potential for contraction of home ranges and for dietary mismatches (Birnie‐Gauvin et al., 2017; Murray, Becker, et al., 2016). Specific nutrients or medications (e.g. vaccinations) could also be integrated into supplemental food for such species to help counter the risks of elevated contact rates and pathogen exposure.

Our analyses also demonstrated mixed support for phylogenetic similarity as a tool to identify species with greater disease risks from provisioning. While home range size and dietary breadth were important predictors of effect sizes for microparasites, both estimates of phylogenetic signal (Pagel's λ and *H* ^2^) were low for this parasite group (0.11 and 0 respectively); this discrepancy could in part reflect the relatively small number of species (Münkemüller et al., 2012). Yet, while effect sizes for helminths and ectoparasites also showed no phylogenetic dependence using λ, we detected demonstrated moderate phylogenetic heritability (0.4 and 0.46). This discordance could reflect λ underestimating true heritability (Vrancken et al., 2015) and that averaging within‐species variance likewise underestimated λ; the small sample size for these parasite datasets could also explain low λ (Münkemüller et al., 2012). Importantly, as effect sizes for helminths were poorly explained by traits but had moderate *H* ^2^, responses to provisioning could be better predicted by host phylogeny. For example, both yellow baboons (*Papio cynocephalus*) and long‐tailed macaques (*Macaca fascicularis*) showed greater helminth infection in provisioned habitats, which suggests other Old World primates could show similar outcomes. Moderate phylogenetic signals could thus motivate future parasite surveillance of species such as vervet monkeys (*Chlorocebus aethiops*), which forage near human settlements and can be infected by several zoonotic helminths (Gillespie, Greiner, & Chapman, 2004).

## 5 CONCLUSIONS

Given the widespread nature of human activities that provision wildlife, understanding the intrinsic trait drivers of how infection responds to supplemental resources is important for conservation and human health and can inform ecological links between resource heterogeneity and host–parasite interactions. Host trait profiles identified here suggest testable hypotheses for future field studies comparing infection outcomes between natural and provisioned populations. Future work across a broader range of taxa will enhance our predictions of which species tend to experience elevated infection by which parasite groups in response to provisioning and will hence increase our ability to manage emerging disease risks to wildlife, domestic animals and humans.

## AUTHORS' CONTRIBUTIONS

D.J.B., D.G.S. and S.A. designed the study; D.J.B. collected data, performed the analyses, and wrote the manuscript, with all co‐authors contributing substantially to revisions.

## DATA ACCESSIBILITY

Data available from the Dryad Digital Repository: https://doi.org/10.5061/dryad.278rt (Becker, Streicker, & Altizer, 2017).
