# Supplementary material for: Using host species traits to understand the consequences of resource provisioning for host–parasite interactions
Source: J Anim Ecol. 2017 Nov 13;87(2):511–25. doi: 10.1111/1365-2656.12765 (PMC5836909; doi:10.1111/1365-2656.12765)
Supplement: Supplementary file 1 [file JANE-87-511-s001.pdf]

Using host species traits to understand the consequences of resource provisioning for host–parasite interactions: Supporting Information

Daniel J. Becker, Daniel G. Streicker, Sonia Altizer

Appendix 1. Effect size data collection
Appendix 2. Correlations among pace of life variables
Appendix 3. Trait data collection
Appendix 4. Correlation between covariates
Appendix 5. Full ranking of candidate MEMs
Appendix 6. Works cited

Appendix 1. Effect size data collection

Systematic searches were performed on February 9, 2017 within the Web of Science, Google Scholar, CAB Abstracts, and PubMed databases, spanning 2015 to 2017. We used the following search string in Web of Science, CAB Abstracts, and PubMed.

("anthropogenic food" OR "anthropogenic resource" OR provisioning OR provisioned OR "supplemental feed" OR "supplemental resource" OR "resource supplementation" OR "resource subsidies" OR "anthropogenic subsidies" OR "human-provided subsidies" OR "human-provided resource" OR "host resource")

AND

(disease* OR infect* OR pathogen* OR bacteria* OR parasite* OR virus* OR helminth* OR protozoa* OR epidemic* OR transmission*)

AND (wildlife OR wild)

Our Google Scholar search contained the following search string.

("anthropogenic food" OR provisioning OR "supplemental feed*" OR "supplemental resource*" OR "resource subsidies*" OR supplementation) AND (disease* OR infect* OR pathogen* OR transm*) AND wildlife

As the goal of our analysis was to examine host traits as predictors of effect sizes, we increased our sample by also including studies referenced in papers included in the quantitative synthesis.

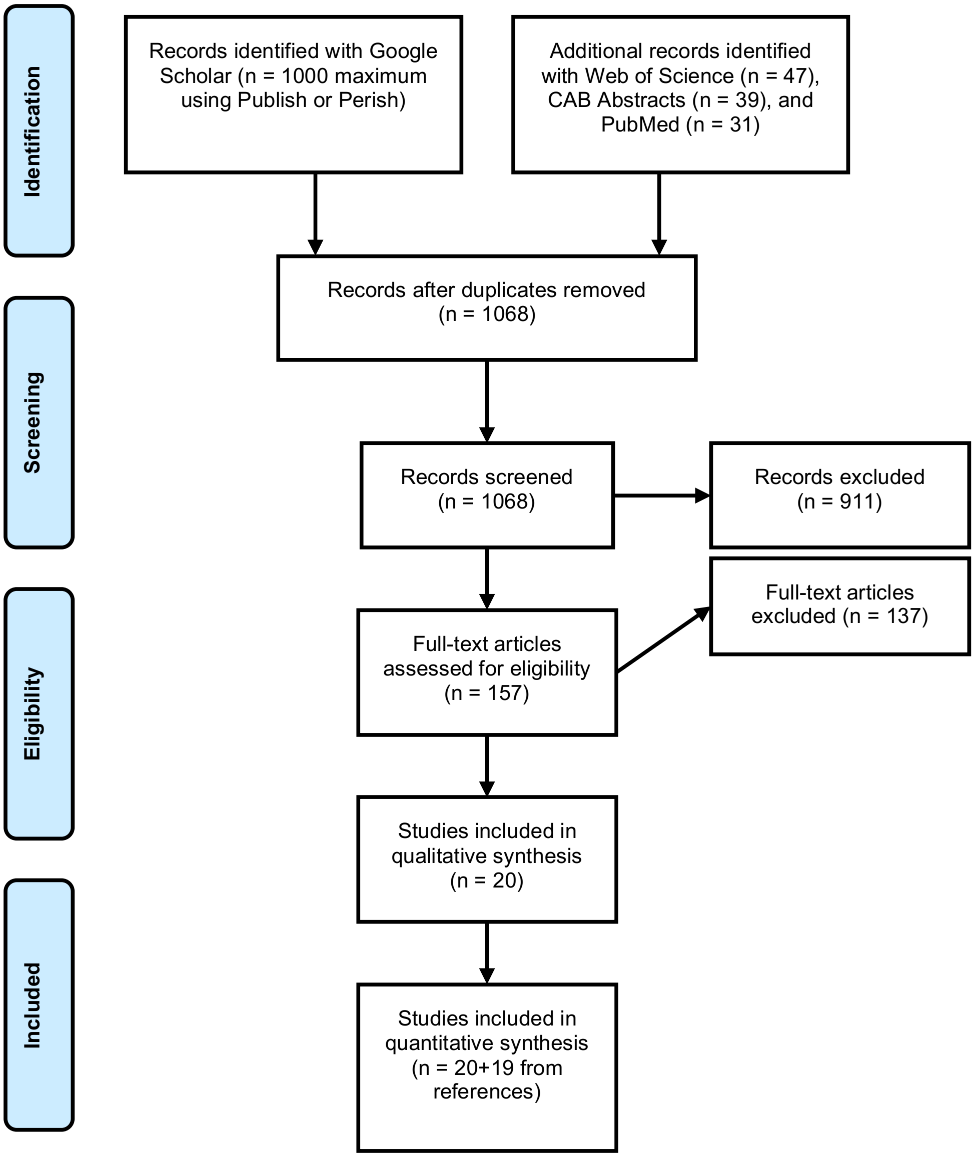

Figure S1. PRISMA diagram documenting the data collection and inclusion process.

Appendix 2. Correlations among pace of life variables

Table S1. Correlation coefficients (*r*) for life history variables built into the phylogenetic PCA.

	log(mass)	sqrt(body size)	log1p(litter size)	log(sex mat)	log(lifespan)
log(mass)	1	–	–	–	–
sqrt(body size)	0.90	1	–	–	–
log1p(litter size)	–0.39	–0.51	1	–	–
log(sex mat)	0.54	0.50	–0.17	1	–
log(lifespan)	0.55	0.58	–0.37	0.67	1

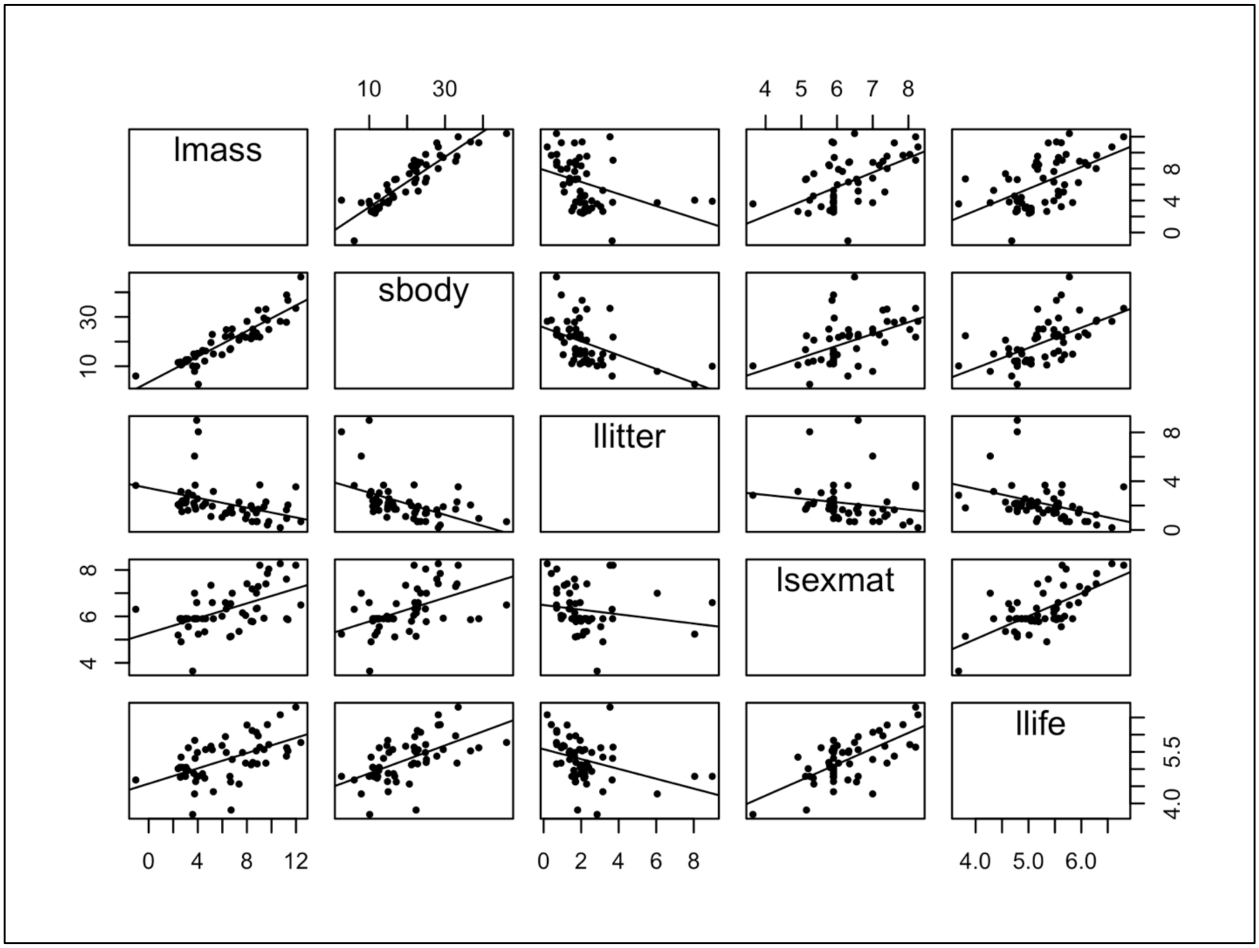

Figure S2. Correlation matrix for life history variables built into the phylogenetic PCA.

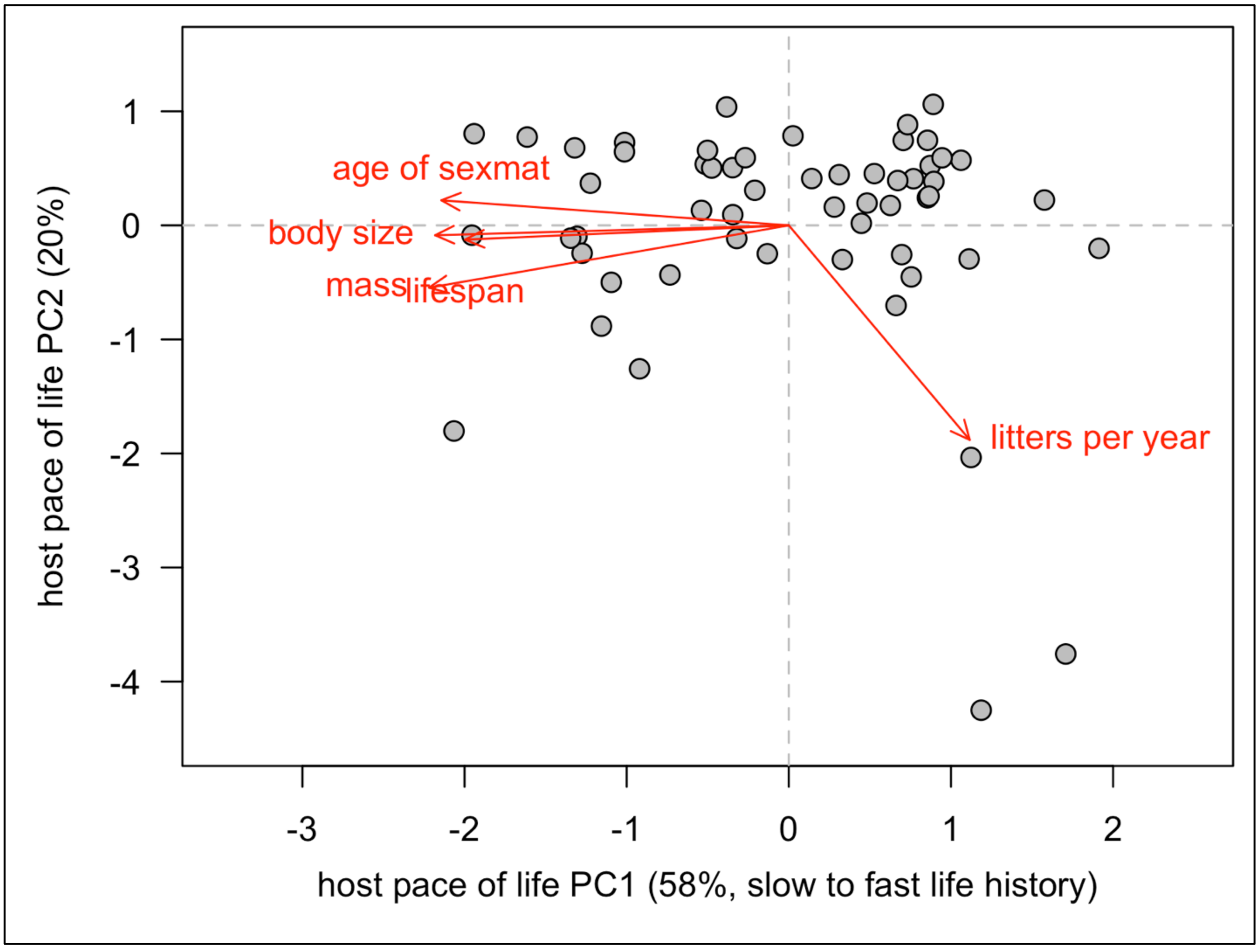

Figure S3. Biplot of the first two phylogenetic PCs for pace of life covariates.

Appendix 3. Trait data collection

As noted in the main text, trait data for non-mammals and for values missing from PanTHERIA (Jones *et al.* 2009) were derived from the AnAge database (http://genomics.senescence.info/species/), Animal Diversity Web at the University of Michigan Museum of Zoology (http://animaldiversity.org/), ARKive (http://www.arkive.org/), or the primary literature. Trait data collection was standardized to match definitions provided by PanTHERIA (Table S1).

Table S2. Trait variable definitions and units

Trait	Definition	Units
Adult body mass	Body mass of adults	Grams
Adult body size	Length from nose/beak/snout to the base of the tail	Millimeters
Litter size	Number of offspring per clutch/litter per female	–
Litters per year	Number of clutches/litters per year	–
Total litters	Litter size * litters per year	–
Lifespan	Maximum recorded longevity	Months
Sexual maturity	Age when individuals are sexually mature or mate	Days
Diet breadth	Number of diet categories consumed per species	–
Trophic level	Herbivore, omnivore, or carnivore	–
Home range	Size of area used for everyday activities	Km^2
Migratory status	Resident or migrant (either partial or full)	–

For diet breadth, eight categories exist in PanTHERIA: vertebrate, invertebrate, fruit, flowers/nectar/pollen, leaves/branches/bark, seeds, grass, and roots/tubers. For trophic level, carnivores were defined as hosts with a diet of vertebrates or invertebrates and herbivores were defined as hosts with a diet of fruit, flowers/nectar/pollen, leaves/branches/bark, seeds, grass, and/or roots/tubers. Species were also categorized as migratory if they exhibited partial migration (e.g., between primary and secondary nurseries for *Dasyatis americana*).

We here outline the data collection process for missing trait values and for non-mammals. We revised all diet breadth and trophic level data from PanTHERIA for consistency, using information from the Animal Diversity Web (ADW).

Dasyatis americana

Missing traits	Range, mean, or items	Final value	Source
Adult body mass	74580	74580	AnAge
Adult body size	750–800	775	ADW
Litter size	4.2	4.2	AnAge
Litters per year	1	1	ADW
Lifespan	216	216	AnAge
Sexual maturity	2008	2008	AnAge
Diet breadth	Vertebrate, invertebrate	2	ADW
Trophic level	Carnivore	Carnivore	ADW
Home range	0.88	0.88	(Corcoran *et al.* 2013)
Migratory status	Partial[1]	Migratory	ADW, ARKive, (McEachran, De Carvalho & Carpenter 2002)

[1]Known to migrate in schools to higher latitudes during summer months

Microtus agrestis

Missing traits	Range, mean, or items	Final value	Source
Adult body size	90–155	102.5	ARKive
Diet breadth	Grass, bark, invertebrate	3	ADW
Trophic level	Omnivore	Omnivore	ADW
Migratory status	Resident	Resident	ARKive, IUCN

Tamias striatus

Missing traits	Range, mean, or items	Final value	Source
Adult body mass	96	96	AnAge
Litter size	5	5	AnAge
Litters per year	1.5	1.5	AnAge
Lifespan	114	114	AnAge
Sexual maturity	187–228	207.5	AnAge
Diet breadth	Fruit, seed, invertebrate, vertebrate (eggs)	4	ADW
Trophic level	Omnivore	Omnivore	ADW
Home range	0.0008–0.006	0.0034	ADW
Migratory status	Residential	Residential	ADW

Cynomys ludovicianus

Missing traits	Range, mean, or items	Final value	Source
Diet breadth	Invertebrate, seed, flower, leaves/bark, roots, grass	6	ADW
Trophic level	Omnivore	Omnivore	ADW
Migratory status	Residential	Residential	ADW

Lepus americanus

Missing traits	Range, mean, or items	Final value	Source
Diet breadth	Flower, leaves/bark, grass	3	ADW
Trophic level	Herbivore	Herbivore	ADW
Migratory status	Residential	Residential	ADW

Pan troglodytes

Missing traits	Range, mean, or items	Final value	Source
Litters per year	0.2	0.2	AnAge
Diet breadth	Vertebrate, invertebrate, fruit, leaves/bark, seed, flower	6	ADW
Trophic level	Omnivore	Omnivore	ADW
Migratory status	Residential	Residential	ADW

Papio cynocephalus

Missing traits	Range, mean, or items	Final value	Source
Adult body size	508–1143	825.5	ADW
Litters per year	0.5	0.5	ADW
Diet breadth	Vertebrates, invertebrates, fruit, leaves, seeds, roots	6	ADW
Trophic level	Omnivore	Omnivore	ADW
Migratory status	Residential	Residential	ADW

Papio anubis

Missing traits	Range, mean, or items	Final value	Source
Adult body size	480–760	620	ADW
Litters per year	1	1	ADW
Sexual maturity	2555–3650	3102.5	ADW
Diet breadth	Vertebrates, invertebrates, fruit, leaves, seeds, roots	6	ADW
Trophic level	Omnivore	Omnivore	ADW
Migratory status	Residential	Residential	ADW

Macaca mulatta

Missing traits	Range, mean, or items	Final value	Source
Diet breadth	Vertebrate, invertebrate, leaves, roots, fruit	5	ADW
Trophic level	Omnivore	Omnivore	ADW
Migratory status	Residential	Residential	ADW

Macaca fascicularis

Missing traits	Range, mean, or items	Final value	Source
Diet breadth	Invertebrate, fruit, leaves, flower	4	ADW
Trophic level	Omnivore	Omnivore	ADW
Migratory status	Residential	Residential	ADW

Saguinus fuscicollis

Missing traits	Range, mean, or items	Final value	Source
Adult body size	200–230	215	(Heymann 1997)
Litters per year	1	1	AnAge
Diet breadth	Invertebrates, fruit, seed, flower/nectar	4	ADW
Trophic level	Omnivore	Omnivore	ADW
Migratory status	Residential	Residential	ADW

Saguinus mystax

Missing traits	Range, mean, or items	Final value	Source
Adult body size	616	616	ADW
Litters per year	1–2	1.5	ADW
Lifespan	240	240	AnAge
Diet breadth	Vertebrates, invertebrates, fruit, flower/nectar	4	ADW
Trophic level	Omnivore	Omnivore	ADW
Migratory status	Residential	Residential	ADW

Cervus elaphus

Missing traits	Range, mean, or items	Final value	Source
Litters per year	0.9	0.9	AnAge
Diet breadth	Leaves, roots/tubers, grass	3	ADW
Trophic level	Herbivore	Herbivore	ADW
Migratory status	Migratory	Migratory	ADW

Odocoileus virginianus

Missing traits	Range, mean, or items	Final value	Source
Litters per year	1	1	AnAge
Diet breadth	Leaves, roots/tubers, grass	3	ADW
Trophic level	Herbivore	Herbivore	ADW
Migratory status	Residential	Residential	ADW

Sus scrofa

Missing traits	Range, mean, or items	Final value	Source
Diet breadth	Vertebrate, invertebrate, roots, seed, fruit	5	ADW
Trophic level	Omnivore	Omnivore	ADW
Terrestriality	Terrestrial	Terrestrial	ADW
Migratory status	Residential	Residential	ADW

Procyon lotor

Missing traits	Range, mean, or items	Final value	Source
Diet breadth	Vertebrate, invertebrate, roots, seed, fruit	5	ADW
Trophic level	Omnivore	Omnivore	ADW
Migratory status	Residential	Residential	ADW

Herpestes javanicus

Missing traits	Range, mean, or items	Final value	Source
Diet breadth	Vertebrate, invertebrate, seed, fruit	4	ADW
Trophic level	Omnivore	Omnivore	ADW
Migratory status	Residential	Residential	ADW

Nyctereutes procyonoides

Missing traits	Range, mean, or items	Final value	Source
Litters per year	1	1	AnAge
Diet breadth	Vertebrate, invertebrate, leaves, roots, seed, fruit	6	ADW
Trophic level	Omnivore	Omnivore	ADW
Migratory status	Residential	Residential	ADW

Vulpes vulpes

Missing traits	Range, mean, or items	Final value	Source
Litters per year	1	1	AnAge
Diet breadth	Vertebrate, invertebrate, fruit	3	ADW
Trophic level	Omnivore	Omnivore	ADW
Migratory status	Residential	Residential	ADW

Vulpes velox

Missing traits	Range, mean, or items	Final value	Source
Litters per year	1	1	AnAge
Diet breadth	Vertebrate, invertebrate, fruit, seed	4	ADW
Trophic level	Omnivore	Omnivore	ADW
Migratory status	Residential	Residential	ADW

Canis latrans

Missing traits	Range, mean, or items	Final value	Source
Litters per year	1	1	AnAge
Diet breadth	Vertebrate, invertebrate, fruit, leaves	4	ADW
Trophic level	Omnivore	Omnivore	ADW
Migratory status	Residential	Residential	ADW

Petrogale penicillata

Missing traits	Range, mean, or items	Final value	Source
Diet breadth	Leaves/bark, seed, root, fruit	4	ADW
Trophic level	Herbivore	Herbivore	ADW
Home range	0.0201–0.0284	0.02425	(Laws & Goldizen 2003)
Migratory status	Residential	Residential	ADW

Trichosurus vulpecula

Missing traits	Range, mean, or items	Final value	Source
Diet breadth	Leaves, shoots, seed, fruit, flower	5	ADW
Trophic level	Herbivore	Herbivore	ADW
Migratory status	Residential	Residential	ADW

Isoodon obesulus

Missing traits	Range, mean, or items	Final value	Source
Adult body size	500.2	500.2	(Driessen & Rose 2015)
Diet breadth	Invertebrates, fruit, seed, plant fibers	4	ADW
Trophic level	Omnivore	Omnivore	ADW
Migratory status	Residential	Residential	ADW

Molothrus ater

Missing traits	Range, mean, or items	Final value	Source
Adult body mass	38–50	44	ADW
Adult body size	160–220	190	ADW
Litter size[1]	0–77	38.5	ADW
Litters per year[1]	1	1	ADW
Lifespan	202.8	202.8	ADW
Sexual maturity	365	365	ADW
Diet breadth	Invertebrate, seed, fruit, vertebrate (eggs)	4	ADW
Trophic level	Omnivore	Omnivore	ADW
Home range	0.045–0.066	0.0555	ADW
Migratory status	Partial	Migrant	Cornell Lab of Ornithology

[1]Values reflect a range of 0–77 eggs per year, as clutches per year are unknown

Passerina cyanea

Missing traits	Range, mean, or items	Final value	Source
Adult body mass	12–18	15	ADW, AnAge
Adult body size	115–130	122.5	ADW
Litter size	3.5	3.5	AnAge
Litters per year	1	1	AnAge
Lifespan	120	120	AnAge
Sexual maturity	365	365	AnAge
Diet breadth	Invertebrate, seed, fruit	3	ADW
Trophic level	Omnivore	Omnivore	ADW
Home range	0.004–0.08	0.042	ADW
Migratory status	Migratory	Migratory	ADW

Cardinalis cardinalis

Missing traits	Range, mean, or items	Final value	Source
Adult body mass	42.6	42.6	AnAge
Adult body size	209–235	222	ADW
Litter size	3	3	AnAge
Litters per year	2	2	AnAge
Lifespan	342	342	AnAge
Sexual maturity	365	365	ADW
Diet breadth	Invertebrate, fruit, seed	3	ADW
Trophic level	Omnivore	Omnivore	ADW
Home range	0.212	0.212	(Halkin & Linville 1999)
Migratory status	Resident	Resident	ADW

Geospiza fuliginosa

Missing traits	Range, mean, or items	Final value	Source
Adult body mass	14	14	ARKive
Adult body size	110	110	(Swash, Still & Lewington 2005)
Litter size	3	3	ARKive
Litters per year[1]	5–10	7.5	(Grant *et al.* 2000)
Lifespan	180–240	210	(Grant 1985)
Sexual maturity[1]	90–160	135	(Gibbs, Grant & Weiland 1984)
Diet breadth	Invertebrate, fruit, seed, flower	4	ARKive, (Boag & Grant 1984)
Trophic level	Omnivore	Omnivore	ARKive
Home range	0.0001– 0.002	0.00105	(Price 1984)
Migratory status	Resident	Resident	ARKive

[1]Values are reported across *Geospiza* species

Pipilo aberti

Missing traits	Range, mean, or items	Final value	Source
Adult body mass	46	46	AnAge
Adult body size	210–230	220	Cornell Lab of Ornithology
Litter size	3	3	AnAge
Litters per year	1.5	1.5	AnAge
Lifespan	103.2	103.2	AnAge
Sexual maturity	365	365	AnAge
Diet breadth	Invertebrate, seed	2	Cornell Lab of Ornithology
Trophic level	Omnivore	Omnivore	Cornell Lab of Ornithology
Home range	0.019	0.019	(Marshall 1960; Laudenslayer 1981)
Migratory status	Resident	Resident	Cornell Lab of Ornithology

Spizella passerina

Missing traits	Range, mean, or items	Final value	Source
Adult body mass	11–15.5	13.25	ADW
Adult body size	127–147	137	ADW
Litter size	4	4	ADW
Litters per year	2	2	ADW
Lifespan	117	117	ADW
Sexual maturity	365	365	ADW
Diet breadth	Invertebrate, seed, grass, fruit	4	ADW
Trophic level	Omnivore	Omnivore	ADW
Home range	0.011–0.031	0.021	(Odum & Kuenzler 1955)
Migratory status	Migratory	Migratory	ADW

Carduelis tristis

Missing traits	Range, mean, or items	Final value	Source
Adult body mass	11–20	15.5	ADW
Adult body size	110–130	120	ADW
Litter size	5	5	AnAge
Litters per year	2	2	AnAge
Lifespan	156	156	AnAge
Sexual maturity	330	330	ADW
Diet breadth	Invertebrate, seed, grass	3	ADW
Trophic level	Omnivore	Omnivore	ADW
Home range	0.00066	0.00066	(DeGraaf & Yamasaki 2001)
Migratory status	Migratory	Migratory	ADW

Carpodacus mexicanus

Missing traits	Range, mean, or items	Final value	Source
Adult body mass	21.4	21.4	AnAge
Adult body size	140	140	ADW
Litter size	4	4	AnAge
Litters per year	3	3	AnAge
Lifespan	139.2	139.2	AnAge
Sexual maturity	365	365	ADW
Diet breadth	Invertebrate, fruit, seed	3	ADW
Trophic level	Omnivore	Omnivore	ADW
Home range	0.00006	0.00006	(Thompson 1960)
Migratory status[1]	Partial	Migratory	ADW, Cornell Lab of Ornithology

[1]Some populations in the eastern United States migrate; short-distance migrant

Passer domesticus

Missing traits	Range, mean, or items	Final value	Source
Adult body mass	25.3	25.3	AnAge
Adult body size	150–170	160	Cornell Lab of Ornithology
Litter size	5	5	AnAge
Litters per year	4	4	AnAge
Lifespan	276	276	AnAge
Sexual maturity	152–365	258.5	AnAge
Diet breadth	Invertebrate, seed, fruit	3	ADW
Trophic level	Omnivore	Omnivore	ADW
Home range	0.000032– 0.0049	0.002466	(Vangestel *et al.* 2010)
Migratory status	Resident	Resident	Cornell Lab of Ornithology

Mimus polyglottos

Missing traits	Range, mean, or items	Final value	Source
Adult body mass	53	53	AnAge
Adult body size	208–255	231	ADW
Litter size	4	4	AnAge
Litters per year	2–4	2–4	ADW
Lifespan	240	240	AnAge
Sexual maturity	365	365	AnAge
Diet breadth	Vertebrate, invertebrate, fruit, seed	4	ADW
Trophic level	Omnivore	Omnivore	ADW
Home range	0.00358	0.00358	(Logan 1987)
Migratory status	Partial	Migrant	ADW, Cornell Lab of Ornithology

Toxostoma curvirostre

Missing traits	Range, mean, or items	Final value	Source
Adult body mass	80.65		AnAge
Adult body size	270	270	Cornell Lab of Ornithology
Litter size	3	3	AnAge
Litters per year	2	2	AnAge
Lifespan	129.6	129.6	AnAge
Sexual maturity	365	365	AnAge
Diet breadth	Invertebrates, fruit, seed	3	ADW
Trophic level	Omnivore	Omnivore	ADW
Home range	0.02–0.045	0.0325	(Poole 1995)
Migratory status	Resident	Resident	Cornell Lab of Ornithology

Dumetella carolinensis

Missing traits	Range, mean, or items	Final value	Source
Adult body mass	23–56	39.5	ADW
Adult body size	210–240	225	ADW
Litter size	4	4	AnAge
Litters per year	2	2	AnAge
Lifespan	130.8	130.8	AnAge
Sexual maturity	365	365	ADW
Diet breadth	Invertebrate, fruit	2	ADW
Trophic level	Omnivore	Omnivore	ADW
Home range	0.002–0.0041	0.00305	ADW
Migratory status	Migratory	Migratory	ADW

Sitta carolinensis

Missing traits	Range, mean, or items	Final value	Source
Adult body mass	20	20	ADW
Adult body size	150	150	ADW
Litter size	8	8	AnAge
Litters per year	1	1	AnAge
Lifespan	120	120	ADW
Sexual maturity	365	365	ADW
Diet breadth	Invertebrate, seed	2	ADW
Trophic level	Omnivore	Omnivore	ADW
Home range	0.1–0.2	0.15	ADW
Migratory status	Resident	Resident	ADW

Poecile atricapilla[1]

Missing traits	Range, mean, or items	Final value	Source
Adult body mass	11	11	ADW
Adult body size	133	133	ADW
Litter size	7	7	AnAge
Litters per year	1	1	ADW
Lifespan	150	150	ADW
Sexual maturity	180	180	AnAge
Diet breadth	Invertebrate, fruit, seed, vertebrate (carrion)	4	ADW
Trophic level	Omnivore	Omnivore	ADW
Home range	0.015–0.053	0.034	ADW
Migratory status	Resident	Resident	Cornell Lab of Ornithology

[1]*Parus atricapillus*

Baeolophus bicolor

Missing traits	Range, mean, or items	Final value	Source
Adult body mass	21	21	ADW
Adult body size	150–170	160	ADW
Litter size	6	6	ADW
Litters per year	1.5	1.5	ADW
Lifespan	156	156	ADW
Sexual maturity	365	365	ADW
Diet breadth	Invertebrate, fruit, seed	3	ADW
Trophic level	Omnivore	Omnivore	ADW
Home range	0.22– 0.28	0.25	(Condee 1970)
Migratory status	Resident	Resident	Cornell Lab of Ornithology

Pica pica

Missing traits	Range, mean, or items	Final value	Source
Adult body mass	178	178	AnAge
Adult body size	450–600	525	ADW
Litter size[1]	6	6	ADW
Litters per year[1]	1	1	ADW
Lifespan	260.4	260.4	AnAge
Sexual maturity	730	730	AnAge
Diet breadth[1]	Invertebrate, seed, fruit, vertebrate (eggs)	4	ADW
Trophic level[1]	Omnivore	Omnivore	ADW
Home range[1]	0.003	0.003	ADW
Migratory status	Resident	Resident	ADW

[1]Values for *Pica hudsonia*

Psittacula echo

Missing traits	Range, mean, or items	Final value	Source
Adult body mass	150–170	160	ARKive
Adult body size	350–420	385	ARKive
Litter size	2	2	ARKive
Litters per year	1	1	ARKive
Lifespan[1]	225.6–408	288.24	AnAge
Sexual maturity[1]	700.8–2007.5	1540.98	(Young *et al.* 2012)
Diet breadth	Leaves, flowers, fruit, seed	4	ARKive
Trophic level	Herbivore	Herbivore	ARKive
Home range[2]	40	40	(Tatayah *et al.* 2007)
Migratory status	Resident	Resident	AKRive

[1]Values span related *Psittacula* species within referenced source

[2]Restricted to an area of less than 40 km^2 in Mauritius

Aquila adalberti

Missing traits	Range, mean, or items	Final value	Source
Adult body mass	3000	3000	AnAge
Adult body size	750–840	795	ARKive
Litter size	1–4	2.5	(del Hoyo *et al.* 2014)
Litters per year	1	1	(del Hoyo *et al.* 2014)
Lifespan	534	534	AnAge
Sexual maturity	1460–1825	1642.5	(del Hoyo *et al.* 2014)
Diet breadth	Vertebrate	1	ARKive
Trophic level	Carnivore	Carnivore	ARKive
Home range	20.85–38.81	29.83	(Fernández *et al.* 2009)
Migratory status	Resident	Resident	

Picoides pubescens

Missing traits	Range, mean, or items	Final value	Source
Adult body mass	21–28	24.5	ADW
Adult body size	145–170	157.5	ADW
Litter size	4	4	ADW
Litters per year	1	1	ADW
Lifespan	142.8	142.8	ADW
Sexual maturity	365	365	ADW
Diet breadth	Invertebrate, fruit, seed, bark	4	ADW
Trophic level	Omnivore	Omnivore	ADW
Home range	0.02–0.12	0.07	ADW
Migratory status	Resident	Resident	Cornell Lab of Ornithology

Larus delawarensis

Missing traits	Range, mean, or items	Final value	Source
Adult body mass	518.5	518.5	AnAge
Adult body size	430–540	485	ADW
Litter size	3	3	AnAge
Litters per year	1	1	AnAge
Lifespan	381.6	381.6	AnAge
Sexual maturity	730	730	AnAge
Diet breadth	Vertebrate, invertebrate, seed, fruit	4	ADW
Trophic level	Omnivore	Omnivore	ADW
Home range	11	11	(Baird 1977)
Migratory status	Migratory	Migratory	ADW

Ciconia ciconia

Missing traits	Range, mean, or items	Final value	Source
Adult body mass	5000–10000	7500	ADW
Adult body size	1000–1150	1075	ADW
Litter size	4–5	4.5	ADW, AnAge
Litters per year	1	1	ADW
Lifespan	312–468	390	ADW, AnAge
Sexual maturity	1460	1460	ADW, AnAge
Diet breadth	Vertebrate, invertebrate	2	ADW
Trophic level	Carnivore	Carnivore	ADW
Home range[1]	10–120	36	(Christensen *et al.* 2008)
Migratory status	Migratory	Migratory	ADW

[1]Median home range for *Ciconia abdimii* as values for *Ciconia ciconia* are unavailable; however, nesting territory for *C. ciconia* was reported as 1 km^2 (Nowakowski 2003)

Eudocimus albus

Missing traits	Range, mean, or items	Final value	Source
Adult body mass	750–1050	900	ADW
Adult body size	560–680	630	Cornell Lab of Ornithology
Litter size	2–4	3	ADW, AnAge
Litters per year	1	1	ADW
Lifespan	196	196	ADW, AnAge
Sexual maturity	1095	1095	ADW
Diet breadth	Vertebrate, invertebrate	2	ADW
Trophic level	Carnivore	Carnivore	ADW
Home range[1]	100–800	450	ADW
Migratory status	Migratory	Migratory	ADW

Colinus virginianus

Missing traits	Range, mean, or items	Final value	Source
Adult body mass	194	194	AnAge
Adult body size	203–247	225	ADW
Litter size	15	15	AnAge
Litters per year	1.5	1.5	AnAge
Lifespan	76.8	76.8	AnAge
Sexual maturity	365	365	AnAge
Diet breadth	Invertebrate, seed, leaves, fruit, flowers	5	ADW
Trophic level	Omnivore	Omnivore	ADW
Home range	0.1–0.6	0.35	ADW
Migratory status	Resident	Resident	ADW

Turdus merula

Missing traits	Range, mean, or items	Final value	Source
Adult body mass	103.2	103.2	AnAge
Adult body size	235–290	262.5	ARKive
Litter size	2.96	2.96	(Zeraoula *et al.* 2016)
Litters per year	2.3–3.1	2.7	(Snow 1958)
Lifespan	261.6	261.6	AnAge
Sexual maturity	365	365	AnAge
Diet breadth	Invertebrates, fruit, seed	3	ARKive
Trophic level	Omnivore	Omnivore	ARKive
Home range	0.00161874–0.00242811	0.002023425	(Snow 1956)
Migratory status	Partial	Migratory	(Lundberg 1985)

Zosterops lateralis

Missing traits	Range, mean, or items	Final value	Source
Adult body mass	12.7	12.7	AnAge
Adult body size[1]	120	120	ADW
Litter size[1,2]	2–4	3	(Horie & Takagi 2012), ADW
Litters per year[1]	2	2	ADW
Lifespan	156	156	AnAge
Sexual maturity	365	365	AnAge
Diet breadth	Invertebrates, fruit, seed	3	(Moeed 1979)
Trophic level	Omnivore	Omnivore	(Moeed 1979)
Home range[2]	0.0004–0.0025	0.00145	(Horie & Takagi 2012)
Migratory status	Partial	Migratory	(Chan 2001)

[1]*Zosterops pallidus*
[2]*Zosterops japonicus daitoensis*

Chelonia mydas

Missing traits	Range, mean, or items	Final value	Source
Adult body mass	160000	160000	AnAge
Adult body size	710–1530	1120	ADW
Litter size	150	150	AnAge
Litters per year	0.17–0.33	0.22	ADW
Lifespan	900	900	AnAge
Sexual maturity	3650	3650	AnAge
Diet breadth	Invertebrate, grass	2	ADW
Trophic level	Herbivore	Herbivore	ADW
Home range	4.09–39.08	21.585	(Seminoff, Resendiz & Nichols 2002)
Migratory status	Migratory	Migratory	ADW

Cyclura cychlura

Missing traits	Range, mean, or items	Final value	Source
Adult body mass	6657–10380	8518.5	(Knapp, Iverson & Owens 2006)
Adult body size	477–620	477	(Knapp *et al.* 2006)
Litter size	4.5–9.8	7.15	(Knapp *et al.* 2006)
Litters per year	1	1	(Alberts 1999)
Lifespan	280.8	280.8	AnAge
Sexual maturity	2920–4380	3650	(Knapp *et al.* 2006)
Diet breadth	Leaves, flower, fruit, shoots	4	ADW, IUCN
Trophic level	Herbivore	Herbivore	ADW, IUCN
Home range	0.0563– 0.3058	0.18105	(Knapp & Owens 2005)
Migratory status[1]	Resident	Resident	(Iverson, Hines & Valiulis 2004)

[1]“Migrates” 30–173 meters to potential nest sites

Varanus varius

Missing traits	Range, mean, or items	Final value	Source
Adult body mass	14000	14000	(Weavers 1988)
Adult body size	200–2000	1100	(Weavers 1988)
Litter size	6–12	9	(Carter 1990)
Litters per year	1	1	(Carter 1990)
Lifespan	176.4	176.4	AnAge
Sexual maturity	1635	1635	(Mendyk 2012)
Diet breadth	Vertebrate, invertebrate	2	(Guarino 2001)
Trophic level	Carnivore	Carnivore	(Guarino 2001)
Home range	0.65	0.65	(Weavers 1993)
Migratory status	Resident	Resident	(Weavers 1993; Guarino 2002)

Pseudacris regilla

Missing traits	Range, mean, or items	Final value	Source
Adult body mass	0.35	0.35	ADW
Adult body size	25–48	36.5	ADW
Litter size	5–70	37.5	ADW
Litters per year	1	1	ADW
Lifespan	108	108	ADW
Sexual maturity	365–730	547.5	ADW
Diet breadth	Invertebrate, algae	2	ADW
Trophic level	Omnivore	Omnivore	ADW
Home range	0.0000000075	0.0000000075	ADW
Migratory status	Residential	Residential	ADW

Osteopilus septentrionalis

Missing traits	Range, mean, or items	Final value	Source
Adult body mass	57	57	ADW
Adult body size	2.5–12	7.25	ADW
Litter size	130–3000	1565	ADW, ARKive
Litters per year[1]	2	2	ADW
Lifespan	120	120	ADW
Sexual maturity	120–255	187.5	ADW
Diet breadth	Vertebrate, invertebrate, algae	3	ADW
Trophic level	Omnivore	Omnivore	ADW
Home range	0.000009–0.000021	0.000015	(McGarrity & Johnson 2010)
Migratory status	Residential	Residential	ADW

[1]Multiple clutches per year

Rana cascadae

Missing traits	Range, mean, or items	Final value	Source
Adult body mass	28–56	42	ARKive
Adult body size	50–75	62.5	ADW
Litter size	425	425	ADW
Litters per year	1	1	ADW
Lifespan			
Sexual maturity	1095	1095	ADW
Diet breadth	Invertebrate	1	ADW
Trophic level	Carnivore	Carnivore	ADW
Home range[1]	0.0000194–0.001028	0.0005237	(Matthews & Pope 1999)
Migratory status	Migratory	Migratory	(Garwood 2009)

[1]Values from *Rana muscosa* due to unknown range of *R. cascadae*

Rana clamitans

Missing traits	Range, mean, or items	Final value	Source
Adult body mass	30–70	50	(Wells 1977)
Adult body size	75–125	100	ADW
Litter size	4000	4000	AnAge
Litters per year	2	2	ADW
Lifespan	120	120	AnAge
Sexual maturity	730	730	AnAge
Diet breadth	Vertebrate, invertebrate, algae	3	ADW
Trophic level	Omnivore	Omnivore	ADW
Home range	0.0000609	0.0000609	(Martof 1953)
Migratory status	Migratory	Migratory	(Lamoureux, Maerz & Madison 2002)

Appendix 4. Correlation between covariates

Table S3. Test statistics and Pagel's λ for associations between host traits across the full species dataset. Significant associations after accounting for phylogenetic dependence are shown in bold.

	Diet	Trophic level	Pace of life	Home range	Migration
Diet	–	–	–	–	–
Trophic level	**$F_{2,53} = 9.63$; $p < 0.01$; $\lambda = 0.41$**	–	–	–	–
Pace of life	$F_{1,54} = 0.80$; $p = 0.38$; $\lambda = 0.40$	$F_{2,53} = 1.34$; $p = 0.27$; $\lambda = 0.00$	–	–	–
Home range	$F_{1,54} = 0.76$; $p = 0.39$; $\lambda = 0.42$	**$F_{2,53} = 4.94$; $p = 0.01$; $\lambda = 0.84$**	$F_{1,54} = 0.90$; $p = 0.35$; $\lambda = 0.00$	–	–
Migration	$F_{2,53} = 1.50$; $p = 0.23$; $\lambda = 0.30$	$F_{2,53} = 0.71$; $p = 0.49$; $\lambda = 0.00$	$F_{1,54} = 0.64$; $p = 0.54$; $\lambda = 0.00$	$F_{2,53} = 1.60$; $p = 0.21$; $\lambda = 0.84$	–

Table S4. Test statistics for associations between provisioning type and trait covariates, with host species included as a random effect in a GLMM with species included as a random effect. Associations were derived from likelihood ratios tests against an intercept-only GLMM.

	Diet	Trophic level	Pace of life	Home range	Migration
Type of provisioning	X^2=0.45, p=0.80	X^2=0.15, p=0.93	X^2=0.30, p=0.58	X^2=0.14, p=0.71	X^2=0.33, p=0.56

Appendix 5. Full ranking of candidate MEMs

Table S5. Ranking of all candidate MEMs for effect sizes with microparasites Models are ranked by ΔAICc alongside the Akaike weights (w_i), residual phylogenetic signal (H^2), number of parameters (k), and pseudo-R^2 statistics ($R^2_{v\text{–}p}$).

**Microparasite outcomes**	**ΔAICc**	w_i	H^2	k	R^2_v	R^2_r	R^2_p
~ ssrange + 1	0	0.12	0	2	0.08	0.02	0.11
~ diet2 + ssrange + diet2:ssrange + 1	0.26	0.11	0	6	0.14	0.08	0.09
~ diet2 + ssrange + 1	0.42	0.1	0	4	0.11	0.05	0.11
~ diet2 + phypc1 + ssrange + 1	1.22	0.07	0	5	0.11	0.06	0.13
~ diet2 + migrate + ssrange + 1	1.3	0.06	0	5	0.13	0.08	0.13
~ phypc1 + 1	1.45	0.06	0	2	0.03	0	0.01
~ migrate + ssrange + 1	1.9	0.05	0	3	0.08	0.03	0.13
~ ptype + ssrange + 1	2.15	0.04	0	3	0.06	0.02	0.11
~ phypc1 + ssrange + 1	2.18	0.04	0	3	0.06	0.01	0.1
~ diet2 + phypc1 + diet2:phypc1 + 1	2.2	0.04	0	6	0.07	0.05	0
~ diet2 + ptype + ssrange + 1	2.65	0.03	0	5	0.09	0.05	0.11
~ migrate + phypc1 + 1	3.24	0.02	0	3	0.03	0.01	0.03
~ phypc1 + ptype + 1	3.63	0.02	0	3	0	0	0.01
~ diet2 + phypc1 + 1	3.95	0.02	0	4	0.02	0	0
~ migrate + ssrange + migrate:ssrange + 1	3.97	0.02	0	4	0.05	0.03	0.11
~ migrate + ptype + ssrange + 1	4.01	0.02	0	4	0.05	0.03	0.13
~ migrate + 1	4.03	0.02	0.26	2	0	0.02	0.11
~ migrate + phypc1 + ssrange + 1	4.1	0.02	0	4	0.05	0.03	0.11
~ phypc1 + trophic + 1	4.11	0.02	0	4	0.01	0	0.02
~ ptype + ssrange + ptype:ssrange + 1	4.34	0.01	0	4	0.02	0.02	0.1
~ phypc1 + ptype + ssrange + 1	4.35	0.01	0	4	0.03	0.01	0.09
~ ptype + 1	4.59	0.01	0.01	2	0	0	0
~ diet2 + 1	4.84	0.01	0	3	0	0	0
~ trophic + 1	5.06	0.01	0	3	0	0.02	0.06
~ diet2 + migrate + phypc1 + 1	5.15	0.01	0	5	0.04	0.03	0.03
~ diet2 + migrate + 1	5.29	0.01	0	4	0.03	0.04	0.04
~ migrate + phypc1 + migrate:phypc1 + 1	5.3	0.01	0	4	0.02	0.01	0.06
~ migrate + phypc1 + ptype + 1	5.43	0.01	0	4	0	0.01	0.03
~ phypc1 + ptype + phypc1:ptype + 1	5.67	0.01	0	4	0	0	0
~ migrate + phypc1 + trophic + 1	5.9	0.01	0	5	0.01	0.01	0.02
~ migrate + ptype + 1	6.02	0.01	0.07	3	0	0.02	0.11
~ diet2 + phypc1 + ptype + 1	6.19	0.01	0	5	0	0	0
~ phypc1 + ptype + trophic + 1	6.29	0.01	0	5	0	0	0.02
~ migrate + trophic + 1	6.93	0	0.01	4	0	0.02	0.07
~ ptype + trophic + 1	6.96	0	0	4	0	0.02	0.07
~ diet2 + ptype + 1	7.03	0	0	4	0	0	0
~ ptype + trophic + ptype:trophic + 1	7.22	0	0	6	0.02	0.08	0.14
~ diet2 + migrate + ptype + 1	7.44	0	0	5	0.01	0.04	0.04
~ migrate + ptype + migrate:ptype + 1	7.97	0	0.02	4	0	0.03	0.12
~ phypc1 + trophic + phypc1:trophic + 1	8.36	0	0	6	0	0	0.02
~ migrate + ptype + trophic + 1	8.76	0	0	5	0	0.03	0.07
~ migrate + trophic + migrate:trophic + 1	10.17	0	0	6	0	0.03	0.06
~ diet2 + ptype + diet2:ptype + 1	11.16	0	0	6	0	0	0

Table S6. Ranking of all candidate MEMs for effect sizes with helminths. Models are ranked by ΔAICc alongside the Akaike weights (w_i), residual phylogenetic signal (H^2), number of parameters (k), and pseudo-R^2 statistics ($R^2_{v\text{–}p}$).

**Helminth outcomes**	**ΔAICc**	w_i	H^2	k	R^2_v	R^2_r	R^2_p
~ phypc1 + ptype + trophic + 1	0	0.16	65.72	4	0	0.04	0.23
~ ptype + 1	0.74	0.11	50.72	2	0	0.06	0.1
~ ptype + trophic + 1	0.83	0.11	66.17	3	0	0.08	0.16
~ phypc1 + ptype + ssrange + 1	0.96	0.1	41.78	4	0.08	0.09	0.25
~ phypc1 + ptype + 1	1.63	0.07	48.45	3	0	0.06	0.15
~ migrate + ptype + trophic + 1	2.56	0.04	68.18	4	0	0.09	0.16
~ diet2 + ptype + 1	2.73	0.04	43.84	3	0	0.04	0.08
~ migrate + ptype + 1	2.81	0.04	50.38	3	0	0.06	0.09
~ ptype + ssrange + 1	2.88	0.04	54.05	3	0	0.06	0.1
~ diet2 + ptype + diet2:ptype + 1	3.64	0.03	54.03	4	0	0	0.03
~ phypc1 + ptype + phypc1:ptype + 1	3.74	0.02	48.52	4	0	0.06	0.16
~ diet2 + phypc1 + ptype + 1	3.85	0.02	47.14	4	0	0.05	0.14
~ migrate + phypc1 + ptype + 1	3.86	0.02	50.29	4	0	0.06	0.15
~ phypc1 + ssrange + 1	4.1	0.02	45.86	3	0	0	0.21
~ diet2 + migrate + ptype + 1	4.74	0.02	44.57	4	0	0.04	0.07
~ diet2 + ptype + ssrange + 1	4.79	0.01	44.59	4	0	0.05	0.08
~ migrate + ptype + ssrange + 1	4.87	0.01	73.74	4	0	0.07	0.09
~ ptype + ssrange + ptype:ssrange + 1	5.1	0.01	74.88	4	0	0.06	0.08
~ migrate + phypc1 + ssrange + 1	5.27	0.01	43.42	4	0	0.02	0.21
~ ssrange + 1	5.36	0.01	41.36	2	0	0.01	0
~ migrate + 1	5.69	0.01	38.52	2	0	0.01	0
~ migrate + ssrange + 1	5.78	0.01	39.53	3	0	0.01	0
~ phypc1 + 1	5.91	0.01	46.89	2	0	0	0.08
~ diet2 + 1	6	0.01	41.86	2	0	0	0
~ trophic + 1	6.09	0.01	43.73	2	0	0	0.04
~ diet2 + phypc1 + ssrange + 1	6.33	0.01	45.76	4	0	0	0.21
~ diet2 + migrate + ssrange + 1	7.04	0	37.28	4	0	0.01	0
~ migrate + phypc1 + migrate:phypc1 + 1	7.18	0	82.17	4	0	0.03	0.07
~ diet2 + ssrange + 1	7.18	0	41.12	3	0	0	0
~ migrate + ssrange + migrate:ssrange + 1	7.56	0	83.49	4	0	0.02	0
~ diet2 + migrate + 1	7.64	0	41.03	3	0	0	0.01
~ migrate + trophic + 1	7.7	0	44.8	3	0	0.02	0.01
~ migrate + phypc1 + 1	7.79	0	46.96	3	0	0	0.04
~ phypc1 + trophic + 1	7.83	0	82.48	3	0	0	0.14
~ diet2 + phypc1 + 1	8.05	0	47.63	3	0	0	0.03
~ diet2 + ssrange + diet2:ssrange + 1	9.31	0	42.45	4	0	0	0
~ migrate + phypc1 + trophic + 1	9.67	0	83.5	4	0	0	0.13
~ phypc1 + trophic + phypc1:trophic + 1	9.8	0	84.26	4	0	0	0.13
~ diet2 + migrate + phypc1 + 1	9.86	0	47.52	4	0	0	0
~ diet2 + phypc1 + diet2:phypc1 + 1	10.04	0	51.34	4	0	0	0.02

Table S7. Ranking of all candidate MEMs for effect sizes with ectoparasites. Models are ranked by ΔAICc alongside the Akaike weights (w_i), residual phylogenetic signal (H^2), number of parameters (k), and pseudo-R^2 statistics ($R^2_{v\text{–}p}$).

**Ectoparasite outcomes**	**ΔAICc**	w_i	H^2	k	R^2_v	R^2_r	R^2_p
~ diet2 + ssrange + 1	0	0.29	0	3	0.52	0.28	0.14
~ diet2 + ptype + ssrange + 1	1.49	0.14	0	4	0.5	0.29	0.14
~ diet2 + migrate + ssrange + 1	2.45	0.09	0	4	0.51	0.28	0.14
~ diet2 + phypc1 + ssrange + 1	2.45	0.09	0	4	0.51	0.28	0.14
~ diet2 + ssrange + diet2:ssrange + 1	2.52	0.08	0	4	0.51	0.28	0.14
~ ssrange + 1	2.77	0.07	0	2	0.49	0.2	0.12
~ diet2 + phypc1 + diet2:phypc1 + 1	3.27	0.06	0	4	0.52	0.23	0.16
~ migrate + ptype + ssrange + 1	4.24	0.03	22.95	4	0.36	0.23	0.12
~ phypc1 + ssrange + 1	4.29	0.03	0.02	3	0.48	0.22	0.12
~ migrate + ssrange + 1	4.3	0.03	0	3	0.48	0.22	0.11
~ ptype + ssrange + 1	4.69	0.03	29.84	3	0.3	0.15	0.11
~ migrate + phypc1 + ssrange + 1	5.62	0.02	25.4	4	0.32	0.24	0.12
~ phypc1 + ptype + ssrange + 1	6.35	0.01	33.2	4	0.26	0.16	0.11
~ migrate + ssrange + migrate:ssrange + 1	6.8	0.01	38.37	4	0.17	0.21	0.11
~ ptype + ssrange + ptype:ssrange + 1	7.07	0.01	35.11	4	0.23	0.12	0.1
~ diet2 + phypc1 + 1	9.68	0	41.33	3	0.11	0.1	0.03
~ phypc1 + 1	10.68	0	0	2	0.42	0.05	0.04
~ diet2 + migrate + phypc1 + 1	12.06	0	51.79	4	0	0.08	0.02
~ diet2 + phypc1 + ptype + 1	12.08	0	51.41	4	0	0.09	0.02
~ phypc1 + trophic + 1	12.45	0	0	4	0.16	0.11	0.05
~ diet2 + 1	12.83	0	53.31	2	0	0	0
~ migrate + phypc1 + 1	12.93	0	56.65	3	0	0.03	0
~ phypc1 + ptype + 1	13.13	0	57.77	3	0	0	0
~ phypc1 + ptype + trophic + 1	13.39	0	0.01	5	0.17	0.09	0.03
~ ptype + 1	13.43	0	56.27	2	0	0	0
~ migrate + 1	13.47	0	53.22	2	0	0	0
~ trophic + 1	13.95	0	55.91	3	0	0.04	0
~ phypc1 + ptype + phypc1:ptype + 1	14.1	0	0.01	4	0.12	0	0
~ diet2 + ptype + 1	14.96	0	59.91	3	0	0	0
~ migrate + phypc1 + trophic + 1	15.05	0	67.33	5	0	0.1	0.04
~ diet2 + migrate + 1	15.26	0	58.94	3	0	0	0
~ migrate + phypc1 + migrate:phypc1 + 1	15.3	0	63.86	4	0	0.02	0
~ migrate + phypc1 + ptype + 1	15.34	0	62.53	4	0	0	0
~ migrate + ptype + 1	15.61	0	60.17	3	0	0	0
~ ptype + trophic + 1	15.94	0	0	4	0.05	0	0
~ migrate + trophic + 1	16.43	0	63.59	4	0	0.02	0
~ diet2 + ptype + diet2:ptype + 1	17.16	0	66.9	4	0	0	0
~ diet2 + migrate + ptype + 1	17.41	0	64.13	4	0	0	0
~ migrate + ptype + trophic + 1	18.57	0	67.72	5	0	0	0

Appendix 6. Works cited

1. Alberts, A. (1999) *West Indian Iguanas: Status Survey and Conservation Action Plan*. IUCN, Gland (Suiza). SSC West Indian Iguana Specialist Group.

2. Baird, P.A. (1977) Feeding Ecology of Ring-Billed and California Gulls (/Larus Delawarensis/ and /L. Californicus/). *Pacific Seabird Group Bulletin*, **4**, 16–17.

3. Boag, P.T. & Grant, P.R. (1984) Darwin's finches (Geospiza) on Isla Daphne Major, Galapagos: breeding and feeding ecology in a climatically variable environment. *Ecological Monographs*, 463–489.

4. Carter, D.B. (1990) Courtship and mating in wild Varanus varius. *Memoirs of the Queensland Museum*, **29**, 333–338.

5. Chan, K. (2001) Partial migration in Australian landbirds: a review, Partial migration in Australian landbirds: a review. *Emu*, **101**, **101**, 281, 281–292, 292.

6. Christensen, K.D., Falk, K., Jensen, F.P. & Petersen, B.S. (2008) Abdim's Stork Ciconia abdimii in Niger: population size, breeding ecology and home range. *Ostrich*, **79**, 177–185.

7. Condee, R.W. (1970) The winter territories of Tufted Titmice. *The Wilson Bulletin*, 177–183.

8. Corcoran, M.J., Wetherbee, B.M., Shivji, M.S., Potenski, M.D., Chapman, D.D. & Harvey, G.M. (2013) Supplemental feeding for ecotourism reverses diel activity and alters movement patterns and spatial distribution of the southern stingray, Dasyatis americana. *PloS one*, **8**, e59235.

9. DeGraaf, R.M. & Yamasaki, M. (2001) *New England Wildlife: Habitat, Natural History, and Distribution*. UPNE.

10. Driessen, M.M. & Rose, R.K. (2015) Isoodon obesulus (Peramelemorphia: Peramelidae). *Mammalian Species*, 112–123.

11. Fernández, M., Oria, J., Sánchez, R., Gonzalez, L.M. & Margalida, A. (2009) Space Use of Adult Spanish Imperial Eagles Aquila adalberti. *Acta Ornithologica*, **44**, 17–26.

12. Garwood, J. (2009) *Spatial Ecology of the Cascades Frog: Identifying Dispersal, Migration, and Resource Uses at Multiple Spatial Scales*. Humboldt State University.

13. Gibbs, H.L., Grant, P.R. & Weiland, J. (1984) Breeding of Darwin's Finches at an Unusually Early Age in an El Niño Year. *The Auk*, **101**, 872–874.

14. Grant, P.R. (1985) Climatic Fluctuations on the Galapagos Islands and Their Influence on Darwin's Finches. *Ornithological Monographs*, 471–483.

15. Grant, P.R., Grant, B.R., Keller, L.F. & Petren, K. (2000) EFFECTS OF EL NIñO EVENTS ON DARWIN'S FINCH PRODUCTIVITY. *Ecology*, **81**, 2442–2457.

16. Guarino, F. (2001) Diet of a large carnivorous lizard, Varanus varius. *Wildlife Research*, **28**, 627–630.

17. Guarino, F. (2002) Spatial ecology of a large carnivorous lizard, Varanus varius (Squamata: Varanidae). *Journal of Zoology*, **258**, 449–457.

18. Halkin, S.L. & Linville, S.U. (1999) Northern Cardinal(Cardinalis cardinalis). *The Birds of North America*, 32.

19. Heymann, E.W. (1997) The relationship between body size and mixed-species troops of tamarins (Saguinus spp.). *Folia Primatologica; International Journal of Primatology*, **68**, 287–295.

20. Horie, S. & Takagi, M. (2012) Nest positioning by male Daito White-eyes Zosterops japonicus daitoensis improves with age to reduce nest predation risk. *Ibis*, **154**, 285–295.

21. del Hoyo, J., Elliott, A., Sargatal, J., Christie, D.A. & de Juana, E. (2014) *Handbook of the Birds of the World Alive. Lynx Edicions, Barcelona*.

22. Iverson, J.B., Hines, K.N. & Valiulis, J.M. (2004) The nesting ecology of the Allen Cays rock iguana, Cyclura cychlura inornata in the Bahamas. *Herpetological Monographs*, **18**, 1–36.

23. Jones, K.E., Bielby, J., Cardillo, M., Fritz, S.A., O'Dell, J., Orme, C.D.L., Safi, K., Sechrest, W., Boakes, E.H., Carbone, C., Connolly, C., Cutts, M.J., Foster, J.K., Grenyer, R., Habib, M., Plaster, C.A., Price, S.A., Rigby, E.A., Rist, J., Teacher, A., Bininda-Emonds, O.R.P., Gittleman, J.L., Mace, G.M., Purvis, A. & Michener, W.K. (2009) PanTHERIA: a species-level database of life history, ecology, and geography of extant and recently extinct mammals. *Ecology*, **90**, 2648–2648.

24. Knapp, C.R., Iverson, J.B. & Owens, A.K. (2006) Geographic variation in nesting behavior and reproductive biology of an insular iguana (Cyclura cychlura). *Canadian Journal of Zoology*, **84**, 1566–1575.

25. Knapp, C.R. & Owens, A.K. (2005) Home range and habitat associations of a Bahamian iguana: implications for conservation. *Animal Conservation*, **8**, 269–278.

26. Lamoureux, V.S., Maerz, J.C. & Madison, D.M. (2002) Premigratory Autumn Foraging Forays in the Green Frog, Rana clamitans. *Journal of Herpetology*, **36**, 245–254.

27. Laudenslayer, W.F. (1981) *Habitat Utilization by Birds of Three Desert Riparian Communities*.

28. Laws, R.J. & Goldizen, A.W. (2003) Nocturnal home ranges and social interactions of the brushtailed rock-wallaby Petrogale penicillata at Hurdle Creek, Queensland. *Australian Mammalogy*, **25**, 169–176.

29. Logan, C.A. (1987) Fluctuations in Fall and Winter Territory Size in the Northern Mockingbird (Mimus polyglottos)(Fluctuaciones en el Tamaño del Territorio de Sinsontes (Mimus polyglottos) durante el Otoño y el Invierno). *Journal of Field Ornithology*, 297–305.

30. Lundberg, P. (1985) Dominance behaviour, body weight and fat variations, and partial migration in European blackbirds Turdus merula. *Behavioral Ecology and Sociobiology*, **17**, 185–189.

31. Marshall, J.T. (1960) Interrelations of Abert and Brown towhees. *The Condor*, **62**, 49–64.

32. Martof, B. (1953) Home Range and Movements of the Green Frog, Rana Clamitans. *Ecology*, **34**, 529–543.

33. Matthews, K.R. & Pope, K.L. (1999) A telemetric study of the movement patterns and habitat use of Rana muscosa, the mountain yellow-legged frog, in a high-elevation basin in Kings Canyon National Park, California. *Journal of Herpetology*, 615–624.

34. McEachran, J.D., De Carvalho, M.R. & Carpenter, K.E. (2002) Batoid fishes. *The living marine resources of the Western Central Atlantic*, **1**, 507–589.

35. McGarrity, M. & Johnson, S. (2010) A radio telemetry study of invasive cuban treefrogs. *Florida Scientist*, **73**, 225–235.

36. Mendyk, R.W. (2012) Reproduction of varanid lizards (Reptilia: Squamata: Varanidae) at the Bronx Zoo. *Zoo biology*, **31**, 374–389.

37. Moeed, A. (1979) Foods of the silvereye (Zosterops lateralis; Aves) near Nelson, New Zealand. *New Zealand Journal of Zoology*, **6**, 475–477.

38. Nowakowski, J.J. (2003) Habitat Structure and Breeding Parameters of the White Stork Ciconia ciconia in the Kolno Upland (NE Poland). *Acta Ornithologica*, **38**, 39–46.

39. Odum, E.P. & Kuenzler, E.J. (1955) Measurement of territory and home range size in birds. *The Auk*, **72**, 128–137.

40. Poole, A.F. (1995) *The Birds of North America: Life Histories for the 21st Century*. American Ornithologists' Union.

41. Price, T.D. (1984) Sexual Selection on Body Size, Territory and Plumage Variables in a Population of Darwin's Finches. *Evolution*, **38**, 327–341.

42. Seminoff, J.A., Resendiz, A. & Nichols, W.J. (2002) Home range of green turtles Chelonia mydas at a coastal foraging area in the Gulf of California, Mexico. *Marine Ecology Progress Series*, **242**, 253–265.

43. Snow, D.W. (1956) Territory in the Blackbird Turdus Merula. *Ibis*, **98**, 438–447.

44. Snow, D.W. (1958) The Breeding of the Blackbird Turdus Merula at Oxford. *Ibis*, **100**, 1–30.

45. Swash, A., Still, R. & Lewington, I. (2005) *Birds, Mammals, and Reptiles of the Galápagos Islands: An Identification Guide*. Yale University Press.

46. Tatayah, R.V.V., Malham, J., Haverson, P., Reuleaux, A. & Van de Wetering, J. (2007) Design and provision of nest boxes for echo parakeets Psittacula eques in Black River Gorges National Park, Mauritius. *Conserv. Evid*, **4**, 16–19.

47. Thompson, W.L. (1960) Agonistic behavior in the House Finch. Part I: Annual cycle and display patterns. *The Condor*, **62**, 245–271.

48. Vangestel, C., Braeckman, B.P., Matheve, H. & Lens, L. (2010) Constraints on home range behaviour affect nutritional condition in urban house sparrows (Passer domesticus). *Biological Journal of the Linnean Society*, **101**, 41–50.

49. Weavers, B.W. (1988) Vital statistics of the lace monitor lizard (Varanus varius) in south-eastern Australia. *Vic Nat*, **105**, 142–145.

50. Weavers, B.W. (1993) Home range of male Lace Monitors, Varanus varius (Reptilia: Varanidae), in south-eastern Australia. *Wildlife Research*, **20**, 303–313.

51. Wells, K.D. (1977) Territoriality and Male Mating Success in the Green Frog (Rana Clamitans). *Ecology*, **58**, 750–762.

52. Young, A.M., Hobson, E.A., Lackey, L.B. & Wright, T.F. (2012) Survival on the ark: life-history trends in captive parrots. *Animal conservation*, **15**, 28–43.

53. Zeraoula, A., Bensouilah, T., Brahmia, H., Bouslama, Z., Houhamdi, M. & Kerfouf, A. (2016) Breeding biology of the European Blackbird Turdus merula in orange orchards. *Journal of King Saud University - Science*, **28**, 300–307.
